# Pregnancy with a prosthetic heart valve, thrombosis, and bleeding: the ESC EORP Registry of Pregnancy and Cardiac disease III

**DOI:** 10.1093/eurheartj/ehaf265

**Published:** 2025-04-16

**Authors:** Johanna A van der Zande, Karishma P Ramlakhan, Karen Sliwa, Justin P Gnanaraj, Hasan Al Farhan, Isabelle Malhamé, Catherine M Otto, Roman Vasallo Peraza, Ariane Marelli, Aldo P Maggioni, Jerome M J Cornette, Mark R Johnson, Jolien W Roos-Hesselink, Roger Hall, the ROPAC investigators, Jolien Roos-Hesselink, Roger Hall, William Parsonage, Werner Budts, Julie de Backer, Jasmin Grewal, Ariane Marelli, Guillaume Jondeau, Mark Johnson, Catherine Otto, Karen Sliwa, Aldo P Maggioni, K Vardanyan, A Melkonyan, H Lachikyan, K Hakobyan, M Mazmanian, H Hayrapetyan, A Tavaracyan, H Poghosyan, R Hovhannisyan, S Sahakyan, S Martirosyan, J Harris, A Pasquet, M Morissens, T Besse-Hammer, B Dumoulin, J De Backer, L Campens, L Demulier, M De Hosson, W Budts, A Van de Bruaene, A Rampelberg, E Troost, L Roggen, P De Meester, J C Mwita, E Tefera, L Kontle, A Marelli, I Malhamé, J Grewal, M Janzen, P A Román Rubio, R Vasallo Peraza, G Vázquez Hernández, J E Pérez Torga, Y Gil Jiménez, M Meluzá Martín, R Almaleh, G Youssef, K Sorour, S Abebe, D Mekonnen, C Fekadu, D Yadeta, S Dupuis-Girod, L Delagrange, M Richardson, L Ghesquiere, O Domanski, M Gonzalez Estevez, Y Ould Hamoud, S Gautier, L Marsili, L Bal-Theoleyre, S Palazzolo, M Ladouceur, G Jondeau, A Bourgeois Moine, L Eliaou, O Milleron, M Tchitchinadze, Y Dulac, C Karsenty, N Souletie, F Bajanca, C Rickers, S Blankenberg, C Sinning, C Magnussen, E Zengin, G Mueller, R Schnabel, Y Von Kodolitsch, R Kozlik-Feldmann, H Baumgartner, R Schmidt, A Hellige, A Rietkötter, M Spartalis, A A Frogoudaki, A Arvanitaki, A Baroutidou, G Giannakoulas, C Karvounis, J P Gnanaraj, A P Steaphen, T Ethirajan, K Kannan, V Subramanian, A Surendran, J Gnanasekaran, V Natarajalingam, S Balasubramani, H Ali Farhan, I F Yaseen, E Mariucci, C Ciuca, F Marchi, G Benedetti, M Baroni, P Festa, A Parlanti, G Scognamiglio, F Fusco, B Sarubbi, M Merlo, B D'Agata Mottolese, C Carriere, G Sinagra, M Bobbo, F Ramani, F M Comoglio, R Bordese, A Pagano, N Montali, V Donvito, C A Remolif, F Petey, B Bouma, S Chamuleau, D Robbers-Visser, T Konings, H Dronkert, D Segers, R Van Kimmenade, H Van Der Zwaan, G Tjalling Sieswerda, A Evers, T Schaap, K Bano, H Yasmeen, K Amir, N Patel, P Akhter, R Khan, A Shakeel, S Mahar, S Habib, M Lelonek, P Hoffman, M Lipczynska, L De Sousa, V Ferreira, T Mano, M Selas, R Cruz Ferreira, E Shlyakhto, O Irtyuga, G Sefieva, K Malikov, T Pervunina, U Shadrina, A Kinsara, D Galzerano, H Al Sergani, W Kurdi, N Kholaif, O Vriz, A Alhamshari, A Alsaigh, O Ahmad, S Alzaher, B Alamro, K Sliwa, F Jakoet-Bassier, L Galian-Gay, A Pijuan-Domenech, B Miranda-Barrio, B Gordon, E Furenäs, J Hlebowicz, F Wedlund, E Nagy, E Mattsson, M Majczuk Sennstrom, P Sörensson, C Christersson, A Lutvica, B Jönelid, T Achter, K Junus, H Gärdesten Wall, M Andreasson, D Tobler, J Bouchardy, F Brand, C Blanche, J Bouchardy, T Rutz, F Brand, U Canpolat, Y Z Sener, N Ozer, E Ayduk Gövdeli, Z Bugra, B Umman, P Karaca Özer, D Baykiz, D Mutlu, H Yalman, B Kilickiran Avci, S Catirli Enar, O Batukan Esen, D Oksen, V Lazoryshynets, S Siromakha, Y Davydova, A Limanska, I Zinovchyk, V Kravchenko, B Kravchuk, O Kravets, N Volkova, O Mazur, O Beregovyi, B T Salih, W A R Al Mahmeed, S Wani, F S Mohamed Farook, G Al Mansoori, S Prakash, R Afifi, D Milewicz, A Cecchi, G Wells, D Sparks, W Wagner, C Bigelow, L Colicchia, T Jentink, M Loichinger, R Saxena, W Wunderlich, C Longtin, P Klopper, R Gobar, J Chou, K Campbell, R Elder, D Halpern, A Hausvater, H Reynolds, N Bhalla, A Small, J Feinberg, P Panday, J Awerbach, J Porche, S Stack, L Mcgrath, A Khan, E Pare, P Woods, C Broberg, K Gibbins, K Brookfield, M Al-Sadawi, A Cove, N Mann

**Affiliations:** Department of Cardiology, Erasmus MC, University Medical Center Rotterdam, Room RG-435, PO Box 2040, 3000 CA Rotterdam, The Netherlands; Department of Obstetrics and Fetal Medicine, Erasmus MC – Sophia Children’s Hospital, University Medical Center Rotterdam, Rotterdam, The Netherlands; Department of Cardiology, Erasmus MC, University Medical Center Rotterdam, Room RG-435, PO Box 2040, 3000 CA Rotterdam, The Netherlands; Department of Obstetrics and Fetal Medicine, Erasmus MC – Sophia Children’s Hospital, University Medical Center Rotterdam, Rotterdam, The Netherlands; Department of Cardiology, Faculty of Health Sciences, Cape Heart Institute, University of Cape Town, Cape Town, South Africa; Institute of Cardiology, Madras Medical College, Chennai, India; Iraqi Board for Medical Specializations, College of Medicine, University of Baghdad, Baghdad Heart Center, Baghdad, Iraq; Department of Medicine, McGill University Health Centre, Montreal, QC, Canada; Division of Cardiology, Department of Medicine, University of Washington School of Medicine, Seattle, WA, USA; Department of Cardiology, Institute of Cardiology and Cardiovascular Surgery, Havana, Cuba; Department of Experimental Medicine, McGill University Health Center, Montreal, QC, Canada; Department of Cardiology, ANMCO Research Center, Florence, Italy; Department of Obstetrics and Fetal Medicine, Erasmus MC – Sophia Children’s Hospital, University Medical Center Rotterdam, Rotterdam, The Netherlands; Department of Obstetric Medicine, Imperial College London, Kensington, London, UK; Department of Cardiology, Erasmus MC, University Medical Center Rotterdam, Room RG-435, PO Box 2040, 3000 CA Rotterdam, The Netherlands; Department of Cardiology, University of East Anglia, Norwich, UK

**Keywords:** Pregnancy, Valve thrombosis, Mechanical valve, Biological valve, Anticoagulation

## Abstract

**Background and Aims:**

Pregnancy in women with a prosthetic heart valve is considered high risk, primarily due to the need for effective anticoagulation. However, data on the relationship between anticoagulation practices and pregnancy outcomes are very limited.

**Methods:**

The Registry of Pregnancy and Cardiac disease is a global registry that prospectively enrolled pregnancies in women with a prosthetic heart valve between January 2018 and April 2023. Detailed data on anticoagulation, including dosage and monitoring, and cardiovascular, pregnancy, and perinatal outcomes were collected.

**Results:**

In total, 613 pregnancies were included of which 411 pregnancies were in women with a mechanical valve and 202 were in women with a biological valve. The chance of an uncomplicated pregnancy with a live birth in women with a mechanical valve was 54%, compared with 79% in women with a biological valve (*P* < .001). Thromboembolic and haemorrhagic complications most frequently occurred when low-molecular weight heparin (LMWH)–based regimens were used. Valve thrombosis occurred in 24 (6%) women, and a prosthetic valve in mitral position was associated with valve thrombosis (odds ratio 3.3; 95% confidence interval 1.9–8.0). A thromboembolic event occurred in 12 (10%) women with anti-Xa monitoring and in 9 (21%) women without (*P* = .060). Foetal death occurred in 20% of all pregnancies.

**Conclusions:**

More favourable outcomes were found in women with a biological valve compared with a mechanical valve. In women with a mechanical valve, the use of LMWH was associated with an increased risk of thromboembolic complications. A mitral prosthetic valve was identified as a predictor for valve thrombosis. The benefit could not be confirmed nor refuted, in terms of reduced thromboembolic events, from using anti-Xa level monitoring in women on LMWH.

## Structured Graphical Abstract

**Structured Graphical Abstract:**
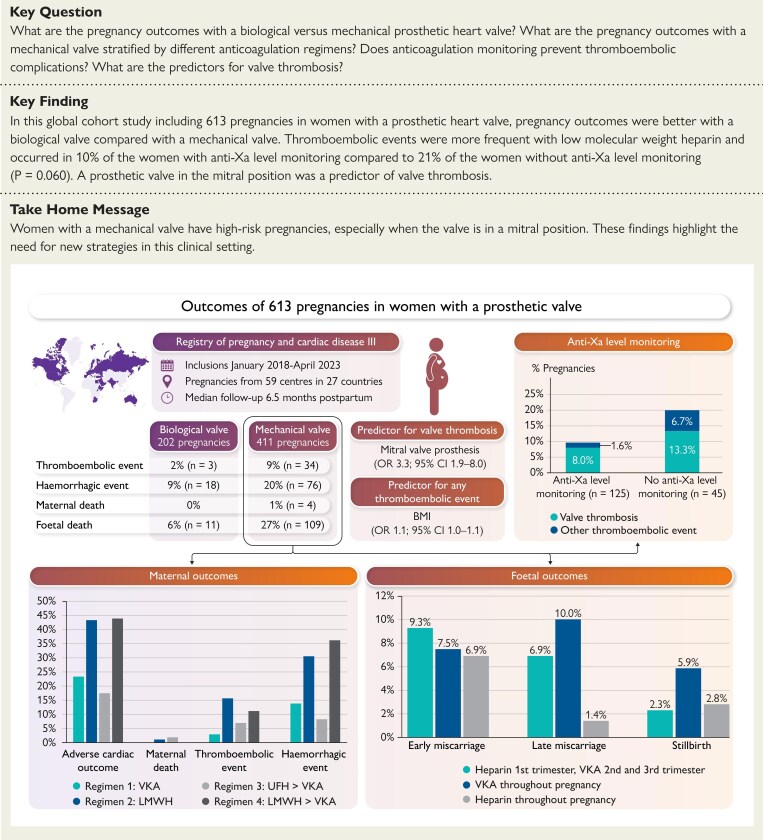

**See the editorial comment for this article ‘The challenges of decision-making in managing women with prosthetic heart valves during pregnancy: a global concern', by J. De Backer**  ***et al*****., https://doi.org/10.1093/eurheartj/ehaf382.**

## Introduction

Cardiovascular disease is a major cause of maternal mortality in pregnant women due to the combination of haemodynamic stress and the hypercoagulable state induced by pregnancy.^1^ The latter is a particular challenge in women with a mechanical valve, in whom a valve thrombosis is associated with a high mortality (20%), but maintaining adequate anticoagulation is associated with a significant bleeding risk.^2^ Indeed, in earlier studies, the chance of experiencing an uncomplicated pregnancy and live birth in women with a mechanical valve was only 58%.^2^ The anticoagulation regimens used during pregnancy in these women differ widely, and all have disadvantages, with vitamin K antagonist (VKA)–based regimen offering a lower maternal risk but higher foetal risk and those based on low-molecular weight heparin (LMWH) or unfractionated heparin (UFH) higher maternal risk but lower foetal risk.^3,4^ The risk of thromboembolic complications is probably lower with adequate anticoagulation achieved by close monitoring, and current guidelines recommend strict international normalized ratio (INR), anti-Xa, and/or activated partial thromboplastin time (aPTT) level measurements. However, the literature on the relationship between these strict measurements and the occurrence of thromboembolic complications is limited. As such, the optimal anticoagulation regimen and how it is monitored are considered to be important knowledge gaps in the latest guidelines on heart disease in pregnancy.^5^ From the outcomes of the Registry of Pregnancy and Cardiac disease (ROPAC) I and II, it was clear that further studies were needed in specific areas, including the management of women with a mechanical heart valve. Therefore, in 2018, ROPAC III was initiated by the European Society of Cardiology (ESC) to prospectively enrol pregnant women with a prosthetic heart valve. We sought to better characterize pregnancy outcomes and compare maternal and perinatal outcomes between different anticoagulation regimens. In addition, since the management of mechanical heart valves in pregnancy is expected to vary depending on resources and local practices, we also aimed for inclusivity of participants from several parts of the globe.

## Methods

The ROPAC III was established in 2018 as a follow-up to ROPAC I–II (2007–17) to exclusively focus on two types of high-risk structural cardiovascular disease: women with a prosthetic valve and women with aortic pathology.^6^ The EURObservational Research Programme (EORP) invited all centres in the ESC, in the Association for European Paediatric and Congenital Cardiology (AEPC), and in ROPAC I–II to participate in ROPAC III. The National Societies and its Working Groups on Congenital Heart Disease and Valve Disease were invited to help in the recruitment of participating centres. Ethical approval or Institutional Review Board approval was obtained according to local legislation. The registry started in January 2018, and pregnancies were included prospectively after obtaining informed consent until April 2023. Inclusion criteria for the ROPAC III were pregnancies in women known to have one or more prosthetic valve and/or known aortic pathology or a genetic condition associated with aortic pathology who were diagnosed before pregnancy. For this current study, we used the data from the women with one or more prosthetic valves.

The pre-pregnancy baseline characteristics and definitions of pregnancy outcomes are shown in Supplementary data online, *Table S1*. Detailed information on anticoagulation use before and throughout pregnancy was recorded, including type of anticoagulation and plan for monitoring, dose of VKA, INR and peak anti-Xa target levels, and INR, anti-Xa, and aPTT level measurements. Anticoagulation use during pregnancy was divided into four regimes: VKA throughout pregnancy (Regimen 1), LMWH throughout pregnancy (Regimen 2), change to UFH early in pregnancy and switch to VKA in second trimester (Regimen 3), and change to LMWH early in pregnancy and switch to VKA in second trimester (Regimen 4). Anticoagulation was switched to LMWH or UFH around 36 weeks of gestation or around delivery in all four regimens. A description of the statistical analyses is presented in Supplementary data online, *Table S2*. The extent of missing values is reported in Supplementary data online, *Table S3*.

## Results

In total, 613 pregnancies in 585 women with a prosthetic heart valve from 59 centres in 27 countries were included in ROPAC III, of which 411 (67%) were in women with a mechanical valve and 202 (33%) were in women with only a biological valve. The pre-pregnancy baseline characteristics are presented in *Table 1*. The mean age during pregnancy was 30.7 ± 5.9 years. The minority was nulliparous (35%). Eighty-one per cent of the pregnancies in women with a mechanical valve were from a low- or middle-income country (LMIC), compared with 38% of the pregnancies in women with a biological valve (*P* < .001).

**Table 1:** Pre-pregnancy baseline characteristics of the total cohort and stratified by type of valve

	Total cohort (*n* = 613)	Mechanical valve (*n* = 411)^a^	Biological valve (*n* = 202)	*P*-value^b^
Pre-pregnancy baseline characteristics				
Age, years, mean ± SD	30.7 ± 5.9	29.9 ± 5.9	32.0 ± 5.6	**<**.**001**
BMI, kg/m^2^, median (Q1–Q3)	24.8 (22.2–28.1)	25.0 (22.5–28.1)	24.7 (21.6–28.8)	.393
Nulliparity	211 (34.6)	141 (34.3)	70 (35.4)	.856
LMIC	409 (66.7)	332 (80.8)	77 (38.1)	**<**.**001**
Current smoker	16 (2.6)	7 (1.7)	9 (4.5)	.059
Chronic hypertension	24 (3.9)	18 (4.4)	6 (3.0)	.509
Atrial fibrillation/flutter	22 (3.7)	16 (4.0)	6 (3.0)	.650
Diabetes mellitus	7 (1.1)	3 (.7)	4 (2.0)	.227
Chronic kidney disease	2 (.3)	1 (.2)	1 (.5)	1.000
Clinical signs of heart failure	40 (6.6)	33 (8.1)	7 (3.5)	.**036**
Estimated SEF < 40%	15 (2.5)	12 (3.1)	3 (1.5)	.286
NYHA class > II	16 (2.7)	14 (3.6)	2 (1.0)	.105
Cyanosis	0 (0)	0 (0)	0 (0)	
Non-cardiac disease	52 (8.5)	33 (8.1)	19 (9.5)	.643
Pre-pregnancy medication use				
Anticoagulation	420 (69.1)	402 (98.5)^c^	18 (9.0)	**<**.**001**
Platelet aggregation inhibitor	86 (14.0)	33 (8.0)	53 (26.2)	**<**.**001**
Other cardiac medication	60 (9.8)	39 (9.6)	21 (10.4)	.773

Data are presented as *n* (%) unless otherwise specified and relate to the number of pregnancies. Bold values denote statistical signifcance at the *P* < .05 level. Percentages are calculated using pairwise deletion. Extent of missing values is reported in Supplementary data online, *Table S3*.

BMI, body mass index; LMIC, low- or middle-income country; NYHA, New York Health Association classification; SEF, systemic ejection fraction.

^a^Nine pregnancies were in women with both a mechanical valve and biological valve during current pregnancy; included in mechanical valve group.

^b^ *P*-value for mechanical valve vs biological valve.

^c^In five pregnancies, the women used no therapeutic anticoagulation before pregnancy for unknown reason; in one pregnancy, the woman only used a daily dose of aspirin of 325 mg.

**Figure 1:**
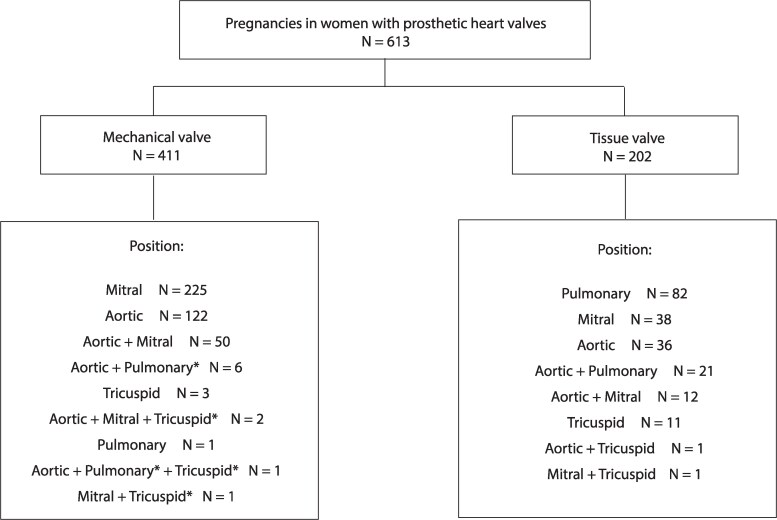
Position of the prosthetic heart valves stratified by type of valve. *N* relates to the number of pregnancies. *The biological valve in women with both mechanical and biological valves (*n* = 9)

The positions of the prosthetic heart valve stratified by type of valve are presented in *Figure 1* and Supplementary data online, *Table S4*. The mean age at first valve replacement was 19.7 ± 8.5 years (*Table 2*). In women with a mechanical valve, rheumatic heart disease was more often the cause of underlying valvular disease (60% vs 18%; *P* < .001). Congenital heart disease (CHD) was more often the cause of underlying valvular disease in women with a biological valve (26% vs 69%; *P* < .001). Prosthetic valve malfunction was more common in women with a biological valve (4% vs 24%; *P* < .001).

**Table 2:** Prosthetic valve details in the total cohort and stratified by type of valve

	Total cohort (*n* = 613)	Mechanical valve (*n* = 411)^a^	Biological valve (*n* = 202)	*P*-value^b^
Age at first valve replacement, years, mean ± SD	19.7 ± 8.5	19.4 ± 7.8	20.2 ± 9.6	.286
Time first valve replacement to current pregnancy, years, mean ± SD	11.0 ± 7.6	10.5 ± 6.9	11.7 ± 8.6^c^	.081
Cause of underlying valvular disease				
Congenital	244 (39.8)	105 (25.5)	139 (68.8)	**<**.**001**
Rheumatic	280 (45.7)	244 (59.4)	36 (17.8)	**<**.**001**
Degenerative	6 (1.0)	4 (1.0)	2 (1.0)	1.000
Other	36 (5.9)	23 (5.6)	13 (6.4)	.716
Multiple causes	30 (4.9)	22 (5.4)	8 (4.0)	.552
Unknown	17 (2.8)	13 (3.2)	4 (2.0)	.450
Malfunction before pregnancy^d^	59 (11.0)	13 (3.8)	46 (23.7)	**<**.**001**
Stenosis	33 (6.2)	9 (2.6)	24 (12.4)	**<**.**001**
Regurgitation	13 (2.4)	3 (.9)	10 (5.2)	.**001**
Stenosis and regurgitation	13 (2.4)	1 (.3)	12 (6.2)	**<**.**001**

Data are presented as *n* (%) unless otherwise specified and relate to the number of pregnancies. Bold values denote statistical significance at the *P* < .05 level. Percentages are calculated using pairwise deletion. Extent of missing values is reported in Supplementary data online, *Table S3*.

^a^Nine pregnancies were in women with both a mechanical valve and biological valve during current pregnancy; included in mechanical valve group.

^b^ *P*-value for mechanical valve vs biological valve.

^c^Forty-nine (24.3%) pregnancies were in women who had a redo biological valve replacement with a mean time between redo valve replacement and current pregnancy of 7.5 ± 5.3 years.

^d^More than mild stenosis and/or regurgitation.

### Maternal outcomes

The maternal outcomes are presented in *Table 3*. An adverse maternal cardiac outcome occurred in 29% of the pregnancies in women with a mechanical valve compared with 19% in women with a biological valve (*P* = .012). Atrial fibrillation/flutter [odds ratio (OR) 12.7; 95% confidence interval (CI) 4.2–38.8], signs of heart failure (OR 3.1; 95% CI 1.2–7.7), and a mechanical prosthetic valve (OR 3.2; 95% CI 1.8–5.7) were associated, and living in an LMIC (OR .4; 95% CI .3–.7) was inversely associated, with an adverse maternal cardiac outcome (see Supplementary data online, *Table S5*). Maternal mortality occurred in four (.7%) pregnancies (see Supplementary data online, *Table S6*). All four women were from a LMIC and had a mechanical valve: two were due to valve thrombosis, one woman suffered sudden cardiac death at home and another woman died 2 days postpartum due to cardiogenic shock. In the latter, a high gradient across the mitral prosthetic valve was observed, but valve thrombosis was not formally diagnosed.

**Table 3:** Maternal outcomes stratified by type of prosthetic valve and type of biological valve

	Stratified by type of prosthetic valve				Stratified by type of biological valve^b^				
	Total (*n* = 613)	Mechanical valve (*n* = 411)^a^	Biological valve (*n* = 202)	*P*-value	Total (*n* = 158)	Homograft (*n* = 49)	Autograft (*n* = 16)	Porcine (*n* = 44)	Bovine (*n* = 49)
Adverse maternal cardiac outcome	152 (25.9)	114 (29.2)	37 (19.0)	.**012**	30 (19.0)	7 (14.3)	2 (12.5)	14 (31.8)	7 (14.3)
Maternal mortality	4 (.7)	4 (1.0)	0 (0)	.307	0 (0)				
Heart failure	37 (6.3)	23 (5.9)	13 (7.1)	.591	12 (7.6)	0 (0)	0 (0)	7 (15.9)	5 (10.2)
Thromboembolic event	37 (6.2)	34 (8.6)	3 (1.5)	**<**.**001**	2 (1.3)	0 (0)	0 (0)	2 (4.5)	0 (0)
Haemorrhagic event	94 (16.1)	76 (19.5)	18 (9.2)	.**002**	3 (1.9)	6 (12.2)	2 (12.5)	5 (11.4)	2 (4.1)
Endocarditis	3 (.5)	3 (.8)	0 (0)	.554	0 (0)				
Arrhythmia	20 (3.4)	13 (3.3)	7 (3.6)	1.000	4 (2.5)	1 (2.0)	0 (0)	3 (7.0)	0 (0)

Data are presented as *n* (%) unless otherwise specified and relate to the number of pregnancies. Bold values denote statistical significance at *P* < .05 level. Percentages are calculated using pairwise deletion. Extent of missing values is reported in Supplementary data online, *Table S3*.

^a^Nine pregnancies were in women with both a mechanical valve and biological valve during current pregnancy; included in mechanical valve group. No differences in maternal outcomes were seen in pregnancies of women with a single tilt disc mechanical valve compared with pregnancies in women with a two tilting discs mechanical valve.

^b^Data on type of biological valve available in 158 out of 202 pregnancies (78%).

Thromboembolic events were more common in women with a mechanical valve compared with a biological valve (9% vs 2%; *P* < .001) and included valve thrombosis in 24 (6%) women with a mechanical valve. In all women with valve thrombosis, at least one echocardiogram was performed and reported with a median of three echocardiograms throughout pregnancy. Details of the valve thrombosis cases including anticoagulation use and anti-Xa, INR, and/or aPTT levels at time of valve thrombosis diagnosis are presented in Supplementary data online, *Table S7*. All valve thromboses occurred during pregnancy at a median gestational age of 19.0 (13.0–29.3) weeks. In 19 women, the mitral valve was thrombosed and in 5 women the aortic valve. Most of the women were on LMWH at time of valve thrombosis diagnosis (*n* = 17), followed by VKA (*n* = 5) and UFH (*n* = 2). In nine (53%) out of 17 women receiving LWMH, the anti-Xa level was reported at diagnosis. In the five women on VKA at time of the occurrence of valve thrombosis, the INR levels were 1.2, 1.2, 1.2, 2.0, and 2.1, respectively, so below the current recommended target levels.^5^ In only one pregnancy complicated by valve thrombosis, a recent (<2 weeks) switch in type of anticoagulation was reported. Details on the cases of other thromboembolic events are presented in Supplementary data online, *Table S8*. The occurrence of a thromboembolic event was 27% in women with a thromboembolic event during a previous pregnancy vs 9% in women without (*P* = .081). For any thromboembolic event during pregnancy, we identified body mass index (BMI) (OR 1.1; 95% CI 1.0–1.1) as independent predictor (see Supplementary data online, *Table S9*). In the univariate logistic regression analysis, we found age (OR 1.1; 95% CI 1.0–1.2) and atrial fibrillation/flutter (OR 3.8; 95% 1.0–14.4) as predictors for valve thrombosis and LMIC (OR .2; 95% CI .1–.5) as a protective predictor (*Table 4*). A prosthetic valve in mitral position (OR 3.3; 95% CI 1.9–8.0) was independently associated with valve thrombosis.

**Table 4:** Predictors of valve thrombosis during pregnancy in women with a mechanical valve

	Univariate model			Multivariate		
	Odds ratio	95% CI lower-upper limit	*P*-value	Odds ratio	95% CI lower-upper limit	*P*-value
Age	1.08	1.01–1.17	.**037**	1.05	.97–1.13	.235
BMI	1.05	.99–1.12	.113			
Nulliparity	1.40	.60–3.23	.436			
LMIC	.21	.09–.49	**<**.**001**	.28	.12–.68	.**005**
Current smoker	2.68	.31–23.20	.372			
Atrial fibrillation/flutter	3.81	1.01–14.37	.**048**	2.78	.73–10.56	.133
Signs of heart failure	1.68	.47–5.96	.421			
SEF < 40%	1.38	.17–11.14	.760			
Non-cardiac disease	2.45	.79–7.65	.123			
Cardiac medication before pregnancy	.40	.05–3.04	.375			
Congenital heart disease	.97	.37–2.51	.949			
Rheumatic heart disease	.56	.24–1.28	.169			
Mitral position	1.70	.71–4.08	.231	3.28	1.87–8.02	.**004**
Aortic position	.46	.15	.160	1.34	.51–3.48	.552
Malfunction of prosthetic valve before pregnancy	1.44	.17	.729			
Plan for anticoagulation level monitoring	.83	.28	.712			

Bold values denote statistical significance at *P* < .05 level. Logistic regression not possible for chronic hypertension, diabetes mellitus, renal disease, and NYHA class > II due to quasi separation.

BMI, body mass index; LMIC, low- or middle-income country; SEF, systemic ejection fraction.

Haemorrhagic events were more frequently seen in pregnancies of women with a mechanical valve (20%) compared with those with a biological valve (9%) (*P* = .002) (*Table 3*). In women with a mechanical valve, haemorrhage during pregnancy occurred in 24 (6%) women, and in 12, details on this haemorrhage were available. Most were in the second trimester (92%) and one occurred in the first trimester. Ten out of these 12 haemorrhages in pregnancy were related to the use of VKAs, of which 6 had an INR value above target level. One woman required blood transfusion. Two women were on LMWH at time of haemorrhage, and both had an anti-Xa level within target level.

Postpartum haemorrhage occurred in 56 (14%) women with a mechanical valve, of which 20 (36%) required treatment. These haemorrhages occurred on average 3.6 ± 1.9 days postpartum.

We found no differences in maternal outcomes in women with a mechanical valve with a single tilting disc and women with a mechanical valve with two tilting discs. The maternal outcomes stratified by type of biological valve are presented in *Table 3*. The maternal outcomes in women with one included pregnancy in the registry and details of the women with ≥2 included pregnancies are presented in Supplementary data online, *Table S10*.

### Obstetric and foetal outcomes

Hypertensive disorders of pregnancy were seen in 20 (3%) women (*Table 5*). Foetal death was reported in 20% of the total cohort. Early miscarriage (9% vs 1%; *P* < .001), late miscarriage (7% vs 0%; *P* < .001), and stillbirth (3% vs 1%; *P* = .043) were more frequently reported in the pregnancies of women with a mechanical valve.

**Table 5:** Obstetric outcomes in the total cohort and stratified by type of valve

	Total (*n* = 613)	Mechanical valve (*n* = 411)^a^	Biological valve (*n* = 202)	*P*-value^b^
Obstetric outcomes				
Hypertensive disorder	20 (3.3)	12 (3.0)	8 (4.0)	.639
PIH	9 (1.5)	6 (1.5)	3 (1.5)	1.000
Pre-eclampsia	9 (1.5)	4 (1.0)	5 (2.5)	.166
Eclampsia/HELLP	2 (.3)	2 (.5)	0 (0)	1.000
Gestational diabetes	21 (3.5)	6 (1.5)	15 (7.5)	**<**.**001**
Foetal outcomes				
Foetal death	120 (20.0)	109 (27.2)	11 (5.5)	**<**.**001**
Early miscarriage (<14 weeks)	36 (6.0)	34 (8.5)	2 (1.0)	**<**.**001**
Late miscarriage (14–23 weeks)	28 (4.7)	28 (7.0)	0 (0)	**<**.**001**
Stillbirth (≥24 weeks)	14 (2.3)	13 (3.3)	1 (.5)	.**043**
Therapeutic abortion	42 (7.0)	34 (8.6)	8 (4.0)	.161
Maternal health	14 (2.3)	12 (3.0)	2 (1.0)	.158
Foetal anomalies	13 (2.2)	9 (2.3)	4 (2.0)	1.000
Psychosocial	15 (2.5)	13 (3.3)	2 (1.0)	.162
Intra-uterine growth restriction	28 (4.6)	11 (2.7)	17 (8.5)	.**003**

	Total (*n* = 533)	Mechanical valve (*n* = 340)^a^	Biological valve (*n* = 193)	*P*-value^b^
Mode of delivery				
Caesarean section	269 (51.6)	178 (53.5)	91 (48.4)	.275
Emergency caesarean section	83 (16.1)	49 (14.8)	34 (18.4)	.124
For cardiac reason	16 (3.1)	15 (4.5)	1 (.5)	.**014**

	Total (*n* = 481)	Mechanical valve (*n* = 292)^a^	Biological valve (*n* = 189)	*P*-value^b^
Neonatal outcomes				
Gestational age at delivery, weeks, median (Q1–Q3)	38.0 (37.0–39.0)	38.0 (37.0–39.0)	38.0 (36.0–39.0)	.364
Preterm birth	107 (22.8)	61 (21.3)	46 (25.0)	.369
Birth weight, g, mean ± SD	2791 ± 604	2737 ± 544	2879 ± 683	.**016**
Small for gestational age	73 (16.4)	50 (18.6)	23 (13.1)	.150
Congenital heart disease	16 (3.5)	5 (1.8)	11 (6.4)	.**016**
Other congenital disease	12 (2.6)	8 (2.9)	4 (2.3)	1.000
Neonatal mortality	5 (1.0)	4 (1.2)	1 (.5)	.658

Data are presented as *n* (%) unless otherwise specified and relate to the number of pregnancies. Bold values denote statistical signficance at *P* < .05 level. Percentages are calculated using pairwise deletion. Extent of missing values is reported in Supplementary data online, *Table S3*.

HELLP, haemolysis, elevated liver enzymes and low platelets syndrome; PIH, pregnancy-induced hypertension.

^a^Nine pregnancies were in women with both a mechanical and biological valve during current pregnancy; included in mechanical valve group.

^b^ *P*-value for mechanical valve vs biological valve.

### Delivery and neonatal outcomes

More than half of the women were delivered by caesarean section (52%) (*Table 5*). The median gestational age at delivery of a live birth was 38.0 (37.0–39.0) weeks with a mean birth weight of 2791 ± 604 g, with a lower mean birth weight in women with a mechanical valve (2737 ± 544 g vs 2879 ± 683 g; *P* = .016). Neonatal mortality was reported in five (1%) pregnancies: two infants died as a result of hydrocephalus; one infant died due to extreme prematurity, sepsis, and multiple organ failure; one infant died due to a septic pneumonia; and another died due to pulmonary haemorrhage. No warfarin embryopathy was reported.

Overall, there was a strikingly different chance of an uncomplicated pregnancy with a live birth between the two valve types: 54% in the women with a mechanical valve and 79% in the women with a biological valve (*P* < .001).

### Low- or middle-income countries vs high-income countries

Baseline characteristics and outcomes compared between pregnancies in women with a prosthetic valve from LMICs and high-income countries (HICs) are presented in *Table 6*. The cause of underlying valvular disease significantly differed: CHD was more common in HICs, and rheumatic heart disease was more common in LMICs. An adverse maternal cardiac outcome occurred more often in women with a mechanical valve from HICs compared with women from LMICs (23% vs 32%; *P* = .029), but no difference in the occurrence of adverse maternal cardiac outcome was seen in women with a biological valve from HICs compared with women from LMICs (17% vs 22%; *P* = .453). Foetal death was more common in women with a mechanical valve from LMICs (30% vs 18% in HICs; *P* = .047).

**Table 6:** Baseline characteristics and outcomes of pregnancies in women with a prosthetic valve from low- or middle-income countries and high-income countries

	Mechanical valve				Biological valve			
	Total (*n* = 411)^a^	LMIC (*n* = 332)	HIC (*n* = 79)	*P*-value^b^	Total (*n* = 202)	LMIC (*n* = 77)	HIC (*n* = 125)	*P*-value^b^
Pre-pregnancy baseline characteristics								
Age, years, mean ± SD	29.9 ± 5.9	29.3 ± 5.9	31.8 ± 5.6	**<**.**001**	32.0 ± 5.6	31.2 ± 6.3	32.6 ± 5.0	.081
Nulliparity	141 (34.3)	103 (31.0)	38 (48.1)	.**005**	70 (35.4)	28 (37.8)	42 (33.9)	.645
Chronic hypertension	18 (4.4)	9 (2.7)	9 (11.4)	.**003**	6 (3.0)	3 (3.9)	3 (2.4)	.677
SEF < 40%	12 (3.1)	10 (3.2)	2 (2.6)	1.000	3 (1.5)	0 (0)	3 (2.4)	.287
NYHA class > II	14 (3.6)	12 (3.8)	2 (2.6)	1.000	2 (1.0)	1 (1.3)	1 (.8)	1.000
Prosthetic valve details								
Age at first valve replacement, years, mean ± SD	19.4 ± 7.8	20.1 ± 7.1	17.1 ± 9.5	.**005**	20.2 ± 9.6	21.1 ± 9.7	19.6 ± 9.5	.289
Time first valve replacement to current pregnancy, years, mean ± SD	10.5 ± 6.9	9.2 ± 5.2	14.8 ± 9.5	**<**.**001**	11.7 ± 8.6	9.8 ± 7.8	12.9 ± 8.9	.**010**
Cause of underlying valvular disease								
Congenital	105 (25.5)	65 (19.5)	40 (50.6)	**<**.**001**	139 (68.8)	36 (46.8)	103 (82.4)	**<**.**001**
Rheumatic	244 (59.4)	223 (67.2)	21 (26.6)	**<**.**001**	36 (17.8)	28 (36.4)	8 (6.4)	**<**.**001**
Other/unknown	62 (15.1)	44 (13.3)	18 (22.8)	.**037**	27 (13.4)	13 (16.9)	14 (11.2)	.289
Anticoagulation regimen^c^				**<**.**001**				
Regimen 1	120 (29.4)	109 (32.9)	11 (14.3)	**<**.**001**				
Regimen 2	72 (17.6)	47 (14.2)	25 (32.5)	**<**.**001**				
Regimen 3	137 (33.6)	136 (41.1)	1 (1.3)	**<**.**001**				
Regimen 4	79 (19.4)	39 (11.8)	40 (51.9)	**<**.**001**				
Maternal outcomes								
Adverse cardiac outcome	152 (25.9)	89 (23.1)	63 (31.5)	.**029**	37 (19.0)	16 (21.9)	21 (17.2)	.453
Maternal mortality	4 (1.0)	4 (1.2)	0 (0)	1.000	0 (0)			
Heart failure	23 (5.9)	15 (4.5)	8 (11.5)	.**029**	13 (7.1)	6 (9.0)	7 (6.0)	.552
Thromboembolic event	34 (8.6)	21 (6.6)	13 (16.5)	.**011**	3 (1.5)	1 (1.3)	2 (1.6)	1.000
Haemorrhagic event	94 (16.1)	47 (12.2)	47 (23.5)	**<**.**001**	18 (9.2)	7 (9.6)	11 (9.0)	1.000
Foetal outcomes								
Foetal death	109 (27.2)	95 (29.5)	14 (17.7)	.**047**	11 (5.5)	6 (7.9)	5 (4.0)	.339
Early miscarriage (<14 weeks)	34 (8.5)	32 (9.6)	2 (2.5)	.**040**	2 (1.0)	0 (0)	2 (1.6)	.526
Late miscarriage (14–23 weeks)	28 (7.0)	23 (6.9)	5 (6.3)	1.000	0 (0)			
Stillbirth (≥24 weeks)	13 (3.2)	13 (3.9)	0 (0)	.083	1 (.5)	0 (0)	1 (.8)	1.000
Therapeutic abortion	34 (8.6)	27 (8.2)	7 (8.9)	.659	10 (5.0)	6 (7.8)^d^	2 (1.6)	.**003**
Intra-uterine growth restriction	11 (2.7)	7 (2.2)	4 (5.1)	.238	17 (8.5)	6 (7.8)	11 (8.9)	.804

	Total (*n* = 333)^a^	LMIC (*n* = 260)	HIC (*n* = 73)	*P*-value^b^	Total (*n* = 193)	LMIC (*n* = 71)	HIC (*n* = 122)	*P*-value^b^
Mode of delivery								
Caesarean section	178 (53.5)	138 (53.1)	40 (54.8)	.894	91 (48.4)	44 (64.7)	47 (39.2)	**<**.**001**

	Total (*n* = 292)^a^	LMIC (*n* = 227)	HIC (*n* = 65)	*P*-value^b^	Total (*n* = 189)	LMIC (*n* = 70)	HIC (*n* = 119)	*P*-value^b^
Neonatal outcomes								
Preterm birth	61 (21.3)	38 (17.0)	23 (37.1)	**<**.**001**	46 (25.0)	20 (29.0)	26 (22.6)	.381
Birth weight, grams, mean ± SD	2737 ± 544	2754 ± 505	2667 ± 683	.294	2879 ± 683	2747 ± 592	2973 ± 688	.**027**
Small for gestational age	50 (18.6)	39 (18.5)	11 (22.0)	1.000	23 (13.1)	10 (16.4)	13 (11.4)	.482
Congenital heart disease	5 (1.8)	3 (1.4)	2 (3.2)	.314	11 (6.4)	9 (15.3)	2 (1.8)	.**001**
Other congenital disease	8 (2.9)	5 (2.3)	3 (4.8)	.382	4 (2.3)	3 (4.9)	1 (.9)	.124
Neonatal mortality	4 (1.2)	4 (1.4)	0 (0)	.579	0 (0)			

Data are presented as *n* (%) unless otherwise specified and relate to the number of pregnancies. Bold values denote statistical significance at *P* < .05 level. Percentages are calculated using pairwise deletion. Extent of missing values is reported in Supplementary data online, *Table S3*.

HIC, high-income country; LMIC, low- or middle-income country; NYHA; New York Heart Association classification; SEF, systemic ventricular ejection fraction.

^a^Nine pregnancies were in women with both a mechanical and biological valve during current pregnancy; included in mechanical valve group.

^b^ *P*-value for LMIC vs HIC.

^c^Regime 1: continue VKA throughout pregnancy. Regime 2: change to LMWH early in pregnancy. Regime 3: change to UFH early in pregnancy and switch to VKA in second trimester. Regime 4: change to LMWH early in pregnancy and switch to VKA in second trimester.

^d^Therapeutic abortion due to foetal anomalies in four pregnancies, compared with zero therapeutic abortions due to foetal anomalies in HICs (*P* = .020).

The chance of an uncomplicated pregnancy with a live birth in women with a mechanical valve was 57% in women from LMICs, compared with 43% in women from HICs (*P* = .033). In women with a biological valve, the chance of an uncomplicated pregnancy with a live birth was 74% in LMICs and 82% in HICs (*P* = .211).

### Anticoagulation regimen

**Figure 2:**
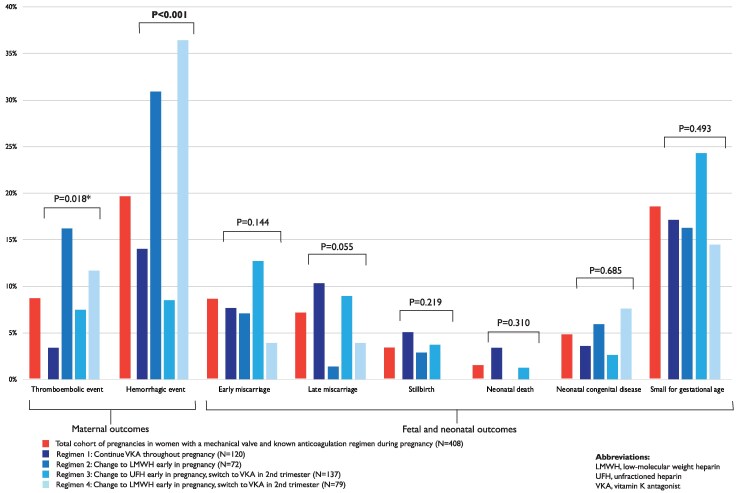
Pregnancy outcomes stratified by anticoagulation regimen. *N* relates to the number of pregnancies. *Not significant with Bonferroni correction. LMWH, low-molecular weight heparin; UFH, unfractionated heparin; VKA, vitamin K antagonist

**Figure 3:**
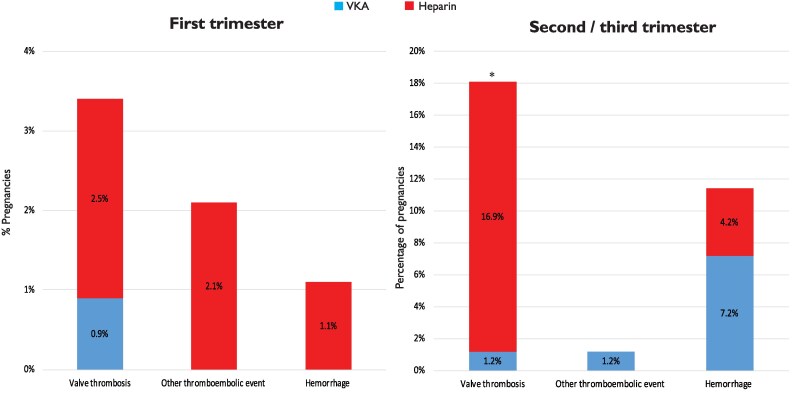
Thromboembolic and haemorrhagic complications stratified by type of anticoagulation and trimester. *P < .001 for VKA compared with heparin. VKA, vitamin K antagonist

Data on anticoagulation use was reported in 408 (99%) of the women with a mechanical valve. Pre-pregnancy characteristics and outcomes stratified by the different anticoagulation regimen are presented in *Figure 2* and Supplementary data online, *Table S11*. The percentages of pregnancies from LMICs and HICs in the different anticoagulation regimens differed markedly (*P* < .001). The vast majority of women in Regimen 1 (91%) and Regimen 3 (99%) were from LMICs. Thromboembolic events were mostly observed in Regimen 2 (16%) and Regimen 4 (12%). Haemorrhagic events occurred most frequently in Regimen 2 (31%) or Regimen 4 (36%), and these haemorrhages mostly occurred postpartum. *Figure 3* shows the thromboembolic and haemorrhagic events stratified by trimester and type of anticoagulation during that event. When heparin was used in the second or third trimester, valve thrombosis occurred more frequently (17%) compared with when VKA was used (1%; *P* < .001).

**Figure 4:**
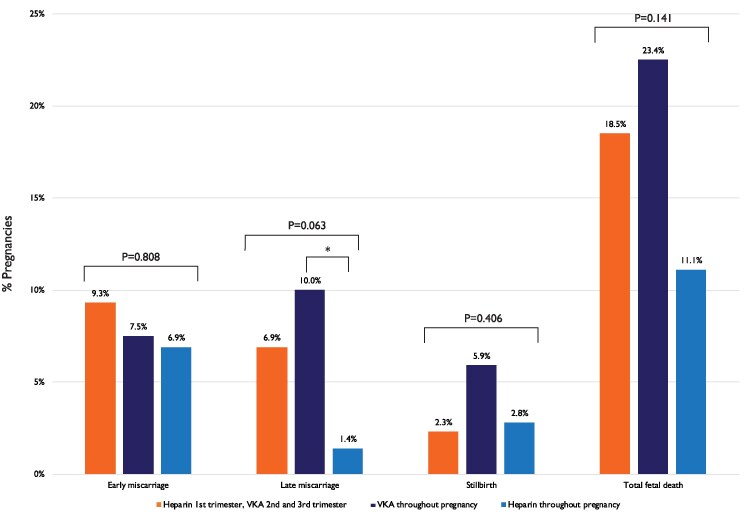
Foetal death in women with a mechanical valve, stratified by anticoagulation regime. VKA, vitamin K antagonist. *P = .034

Foetal death was the highest in Regimen 3 (42%). We found no significant differences between the anticoagulation regimens and the outcomes neonatal death, neonatal congenital disease, and small for gestational age. *Figure 4* shows the foetal death rate in the anticoagulation regimens with a temporary switch to heparin (both LMWH and UFH), VKA throughout pregnancy and heparin throughout pregnancy. We found a higher rate of late miscarriage when VKA was used throughout pregnancy compared with heparin throughout pregnancy (10% vs 1%; *P* = .034). A trend was seen, but significance was not reached, for the differences in total foetal death between VKA throughout pregnancy and heparin throughout pregnancy (23% vs 11%; *P* = .055).

### Anticoagulation level monitoring

**Figure 5:**
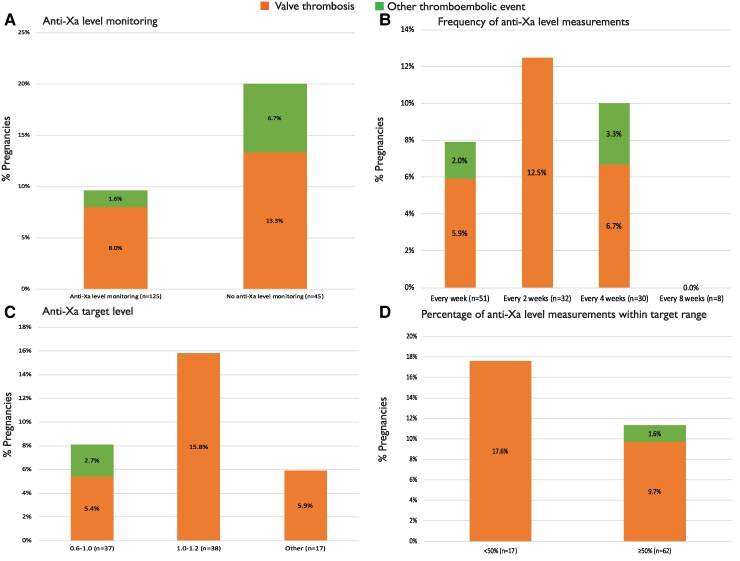
Anti-Xa level monitoring and the occurrence of thromboembolic events. *N* relates to the number of pregnancies

In total, 170 women with a mechanical valve received therapeutic LMWH at some point during pregnancy or postpartum and a plan for anti-Xa monitoring was made in 125 (74%) (*Figure 5A*). A thromboembolic event occurred in 12 (10%) women with a plan for anti-Xa monitoring and in 9 (21%) women without a plan for anti-Xa monitoring (*P* = .060). We found no differences between various frequencies of testing (*P* = .811) (*Figure 5B*) and between different target anti-Xa levels (*P* = .504) (*Figure 5C*). The number of anti-Xa level measurements during pregnancy and postpartum was available in 73 women, and the number of measurements per woman varied between 1 and 50. The median number of anti-Xa level measurements did not differ between women who had a thromboembolic event and women who had not (5.5 vs 5.0 measurements; *P* = .853). Of all anti-Xa level measurements, 71% were within target level. We found no difference in the occurrence of a thromboembolic event between women with <50% and ≥50% of the anti-Xa level measurements within target level (18% vs 11%, respectively; *P* = .814) (*Figure 5D*). Of the women with a thromboembolic event on LMWH, 35% of the anti-Xa level measurements were below the target range compared with 28% in the women without thromboembolic event (*P* = .549). Anti-Xa level monitoring was more often performed in pregnancies in women from HICs compared with women from LMICs (83% vs 48%; *P* < .001).

**Figure 6:**
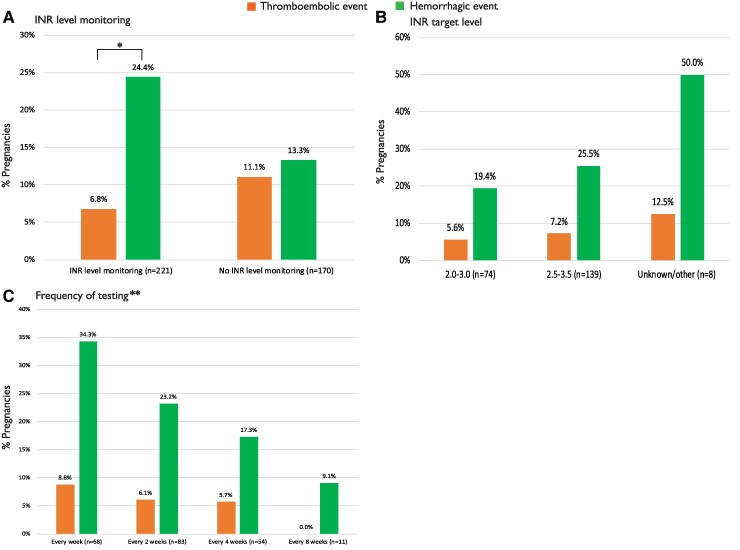
International normalized ratio level monitoring and the occurrence of thromboembolic and haemorrhagic events. *N* relates to the number of pregnancies. INR, international normalized ratio. *P = .008. **P-value for every week vs every 2 weeks: .147; *P*-value for every week vs every 4 weeks: .060; *P*-value for every week vs every 8 weeks: .158

In 391 out of 411 (95%) pregnancies in women with a mechanical valve, VKA was used at some point during pregnancy or postpartum. In 221 (57%) of these women, a plan for INR monitoring was made (*Figure 6A*). We found no differences in the occurrence of a thromboembolic event between women with and without a plan for INR level monitoring (7% vs 11%; *P* = .197), but differences in the occurrence of a haemorrhagic event were seen, with more haemorrhagic events seen with INR level monitoring (24% vs 13%; *P* = .008). We found no differences in thromboembolic event rates and haemorrhagic event rates between different INR target levels (*P* = .585 and *P* = .127, respectively) and between various frequencies of testing (*P* = .827 and *P* = .108, respectively) (*Figure 6B* and *C*). A plan for INR monitoring was made more often in HICs compared with LMICs (78% vs 52%; *P* < .001).

### Dose of vitamin K antagonists and foetal outcomes

The average mean daily warfarin-equivalent dose during pregnancy was 4.7 ± 1.6 mg. Early miscarriage was most frequently seen when the average warfarin-equivalent daily dose was ≥6 mg (21%), while late miscarriage and stillbirth were more commonly reported in the average warfarin-equivalent daily dose of 4.0–4.9 mg (24% and 17%, respectively) (*Table 7*). No difference was observed in neonatal congenital disease between the average warfarin-equivalent daily doses.

**Table 7:** Foetal and neonatal outcomes of pregnancies in women with a mechanical valve who used vitamin K antagonist throughout pregnancy and information on average daily dose was reported, stratified by average warfarin-converted daily dose

	Total^a^ (*n* = 118)	VKA dose < 4.0 mg (*n* = 31)	VKA dose 4.0–4.9 mg (*n* = 29)	VKA dose 5.0–5.9 mg (*n* = 39)	VKA dose ≥ 6 mg (*n* = 19)
Foetal death	30 (25.9)	4 (13.3)	12 (41.4)	6 (15.4)	9 (44.4)
Early miscarriage (<14 weeks)	9 (7.6)	2 (6.5)	0 (0)	3 (7.7)	4 (21.1)
Late miscarriage (14–23 weeks)	12 (10.2)	1 (3.2)	7 (24.1)	3 (7.7)	1 (5.3)
Stillbirth (≥24 weeks)	6 (5.1)	1 (3.2)	5 (17.2)	0 (0)	0 (0)
Therapeutic abortion	3 (2.5)	0 (0)	0 (0)	0 (0)	3 (15.8)^b^

	Total (*n* = 86)	VKA dose < 4.0 mg (*n* = 26)	VKA dose 4.0–4.9 mg (*n* = 17)	VKA dose 5.0–5.9 mg (*n* = 33)	VKA dose ≥ 6 mg (*n* = 10)
Congenital heart disease	1 (1.2)	0 (0)	1 (6.7)	0 (0)	0 (0)
Other congenital disease	2 (2.4)	0 (0)	0 (0)	1 (3.0)	1 (10.0)

Data are presented as *n* (%) unless otherwise specified and relate to the number of pregnancies. Percentages are calculated using pairwise deletion unless otherwise specified. Extent of missing values is reported in Supplementary data online, *Table S3*.

VKA, vitamin K antagonist.

^a^Data on the daily dose was available in 98% (*n* = 108) of the pregnancies in women who used VKA throughout pregnancy. Eighty-three per cent were on warfarin, 15% on acenocoumarol, and 2% on fenprocoumon.

^b^Therapeutic abortion for maternal health issues (*n* = 1) or foetal anomalies (*n* = 2).

### Follow-up

In 425 (70%) women, follow-up data were available. The median follow-up was 6.5 (4.6–9.4) months. An adverse maternal cardiac outcome occurred in 19 (8%) women with a mechanical valve and 6 (3%) women with a biological valve (*P* = .093) (*Table 8*). Two women with a mechanical valve died during follow-up: one woman died 2 months postpartum due to valve thrombosis after she skipped anticoagulation for 3 days, and one woman died 3 years postpartum due to septic shock not related to her cardiac status. Nine (2%) haemorrhagic events occurred, all in women with a mechanical valve. Eight of these haemorrhagic events required treatment and occurred during the first month postpartum.

**Table 8:** Major adverse cardiac event during a median follow-up of 6.5 (4.6–9.4) months

	Total cohort (*n* = 425)	Mechanical valve (*n* = 249)	Biological valve (*n* = 176)	*P*-value
Adverse maternal cardiac outcome	25 (5.9)	19 (7.6)	6 (3.4)	.093
Maternal mortality	2 (.5)	2 (.8)	0 (0)	.514
Heart failure	8 (1.9)	4 (1.6)	4 (2.3)	.723
Thromboembolic event	1 (.2)	1 (.4)	0 (0)	1.000
Haemorrhagic event	9 (2.1)	9 (3.6)	0 (0)	.**009**
Arrhythmia	4 (.9)	2 (.8)	2 (1.1)	1.000

Data are presented as *n* (%) and relate to number of pregnancies. Bold values denote statistical significance at *P* < .05 level.

## Discussion

This is the largest cohort of pregnant women with a prosthetic heart valve collected prospectively so far, and most importantly, detailed information is available on the use and monitoring of anticoagulation during pregnancy. We found more favourable pregnancy outcomes in women with a biological valve. The risk of valve thrombosis in women with a mechanical valve remains very high and is relatively unchanged from what has been previously reported. We now identified a mitral valve prosthesis as a predictor for valve thrombosis. We found no convincing evidence of a significant difference in the occurrence of thromboembolic events between women with or without monitoring of anti-Xa levels while receiving LMWH (*Structured Graphical Abstract*).

Our study confirms that pregnancy in women with a mechanical valve is an extremely challenging situation, with the chance of an uncomplicated pregnancy of only 54%. This is even lower than the 58% we reported in the ROPAC I–-II paper, based on a separate cohort of 212 women with a mechanical valve, and shows that there has been no improvement in outcome in the 8 years between the studies.^2^ The thromboembolic and haemorrhagic event rate in our study was 9% and 20%, respectively, comparable with the previous ROPAC study and with the results of a prospective population-based study from the UK containing 58 women with a mechanical valve.^2,7^ The switch from VKA to heparin and vice versa is recognized as a critical period with a high risk of thromboembolic complications.^8^ However, in our cohort, a recent switch was reported in only one of the cases of valve thrombosis, which may represent underreporting. On the other hand, we all know that this is a critical period; the fall of complications at this point may be due to increased vigilance induced by previous reports. The present results highlight that the risk of valve thrombosis persists throughout pregnancy and that caution is warranted even during periods without switching and that women with a mitral prosthetic valve are at particularly high risk. Not only transthoracic (TTE) but also transoesophageal echocardiography (TEE) should be considered in especially high-risk women at the end of the second or third trimester and in the case of a clinical suspicion of valve thrombosis.^9^ Looking in more detail at the haemorrhagic events, most of these events occurred postpartum. It is unknown if the major postpartum haemorrhages occurred during the switch from heparin back to VKA. However, the timing of these events (mean postpartum day 3.6) does suggest this and reflects a time when both heparin is being used at the same time as the VKA is being re-introduced. Double therapy at a time when damaged tissue has not healed completely causes re-bleed if anticoagulation is excessive. Current ESC pregnancy guidelines and ESC guidelines on valvular heart disease do not contain recommendations regarding the timing and manner of switching back from heparin to VKA after the delivery.^5,10^ It is not inconceivable to postpone the switch from heparin back to VKA until 7–14 days postpartum, when the wound area had already largely healed.

### Biological valves

In line with the previous literature, we found more favourable pregnancy outcomes in the 202 women with a biological valve compared with the women with a mechanical valve,^2^ and these women had a much higher chance of a pregnancy without serious adverse events and a live birth of 79%. We found more pre-pregnancy valvular malfunction (i.e. more than mild) in women with a biological valve compared with women with a mechanical valve, but this did not lead to more adverse maternal cardiac outcomes. These data draw attention to the important topic of choice of valve when replacement is required in women of reproductive age. It is known that biological valves deteriorate over time and probably faster in young patients, requiring re-intervention. Nevertheless, it might be wise to advise a biological valve. However, there are differences in the availability of valves worldwide, with biological valves mainly used in HICs, reflected in the results of the current study. The similar outcomes in women with a biological valve from HICs and LMICs are reassuring, but may reflect the existence of a well-organized healthcare system in those LMICs with some experience of biological valves. Future research should determine whether pregnancy outcomes are improved if more biological valves are placed in young patients in both LMICs and HICs.

### Anticoagulation regimen during pregnancy

The main problem is the need to maintain effective anticoagulation throughout pregnancy without jeopardizing the mother or baby. Both the current ESC pregnancy guidelines and ACC/AHA guidelines for the management of patients with valvular heart disease recommend dose-adjusted LMWH during the first trimester, followed by VKA during the second and third trimesters^5,11^ in women with a pre-pregnancy warfarin-equivalent daily dose of ≥5 mg. When LMWH is unavailable, UFH can be administered during the first trimester, or VKA can be continued in women who require a daily dose of warfarin ≤ 5 mg. As seen in the current study, anticoagulation regimens vary considerably and there seems to be clear preferences for a specific regime in the different parts of the world. We found that Regimen 1 and Regimen 3 were almost exclusively applied in LMICs. Most likely, this is related to the difficulties of anti-Xa monitoring, as also illustrated by the observed lower rates of anti-Xa level monitoring in LMICs compared to HICs. The variation in anticoagulation regimens between countries seems to be a result of differences in accessibility to care and available medical resources. However, we cannot conclude that the regimens used in LMICs were suboptimal. In fact, the outcomes in HICs are worse for thromboembolic and haemorrhagic events. One area of agreement appears to be the way anticoagulants are used around the time of delivery, as everybody seems to follow the guidelines in switching from VKA to a form of heparin. A recent study investigated novel anticoagulation regimens (i.e. a combination of LMWH and low-dose warfarin) in the first trimester in women with a mechanical valve requiring high-dose warfarin and showed promising results.^9^ Future research is needed to determine whether these new regimens could play a role in reducing pregnancy risks for women with a mechanical valve.

### Anticoagulation level monitoring

It is generally accepted that there is uncertainty about whether anti-Xa monitoring is of any value. In non-pregnant patients using LMWH, normal practice is not to monitor anti-Xa levels as previous studies have shown a lack of correlation between effectiveness and the results of monitoring, with an exception for patients who are extremely overweight or have impaired renal function.^12^ The LMWH treatment of pregnant women with mechanical valves has become increasingly prevalent,^13^ particularly in HICs. There have been no controlled trials due to consent issues to show whether or not monitoring to allow dose adjustment is beneficial. A retrospective study, including 155 pregnancies, found more valve thrombosis in women on LMWH without anti-Xa monitoring compared with women who were monitored, but the difference was not significant.^14^ Despite the lack of data, the current pregnancy guidelines recommend weekly anti-Xa level monitoring in pregnant women on LMWH^5^ and recommend that if weekly anti-Xa level monitoring and dose-adjustment is not available, LMWH should be avoided. It should be stressed that these recommendations are based on expert opinion and not on high-quality data. Despite this recommendation, in 26% of the women in the current study who used therapeutic LMWH during pregnancy, anti-Xa levels were not measured. We found a higher rate of thromboembolic events in women without a plan for anti-Xa level monitoring (21%) compared with women with a plan for monitoring (10%); however, statistical significance was not reached. This may have been because of an absence of any benefit or due to small numbers. Possibly in favour of anti-Xa monitoring was the fact that of the women who developed valve thrombosis, 78% of them had anti-Xa levels below the predefined target range at around the time of thrombosis. We do not, however, know whether these were peak levels taken near the time of thrombosis or whether they were random levels taken at the time when thrombosis was detected, or a mix of both. Because of the design of ROPAC as a registry, the data gathered are purely observational and therefore do not allow a scientifically valid comparison between monitoring and its absence. Therefore, any conclusions drawn from ROPAC about the value of anti-Xa level monitoring must be regarded with caution. However, these data do not show any disadvantages of anti-Xa level monitoring, and a reasonable current position would be to continue to follow the current guidelines, unless more definitive data appears.

The higher number of haemorrhagic events in women with a plan for INR level monitoring compared with the women without is remarkable, and the differences in haemorrhagic events are stratified by frequency of testing: the more frequent monitoring, the more haemorrhage slightly counter intuitive (*Figure 6*). An explanation could be that more frequent monitoring leads to more dose adjustments of VKA, which can cause the INR level to rise too high, increasing the risk of bleeding. Conversely, we found too low INR levels in all five women who had valve thrombosis while on VKA. This suggests that, if INR value is within target range, valve thrombosis on VKA could be prevented, and therefore, strict INR level monitoring should be recommended. However, caution is advised with any dose adjustments to avoid overshooting the INR value and the associated increased risk of haemorrhage.

### Vitamin K antagonists throughout pregnancy

Discussion is still ongoing if indeed a lower dose of warfarin is associated with better foetal outcomes. The British Society for Haematology stated in their guideline for anticoagulant management of pregnant individuals with mechanical heart valves that there is insufficient evidence to recommend that lower doses of warfarin are safe in terms of adverse foetal outcomes.^5,15^ Also, our previous ROPAC paper showed no differences in foetal outcomes between a daily dose < 5 mg and ≥5 mg.^2^ To our knowledge, the current study is the first prospective study which investigated a wider range of VKA dose categories beyond just low-dose (<5 mg) and high-dose (≥5 mg). Our data suggest that foetal risks, even with a warfarin-equivalent daily dose of 4.0–4.9 mg, are not negligible. Therefore, we must conclude that a daily warfarin-equivalent dose of <5 mg cannot be considered low risk. However, the lowest risk of overall foetal death was observed in the women with a warfarin-equivalent daily dose < 4.0 mg (*Table 7*). Therefore, it may be worth considering an adjustment to the guidelines to recommend an average daily dose of <4 mg as safer, provided the target INR value is maintained.

### Low- or middle-income countries and high-income countries

We found a lower chance of an event-free pregnancy with a live birth of 43% in HICs vs 57% in LMICs. The anticoagulation strategy in LMICs focused predominantly on the use of warfarin and UFH, and we found that these regimens had better thromboembolic and haemorrhagic outcomes. On the other hand, the foetal death rate is higher in LMICs, and it is worth noting that all maternal deaths were from LMICs. Given the multidisciplinary team and large infrastructure required in the setting of valve thrombosis,^16^ a higher case fatality for valve thromboses in LMIC may be attributed to complex and multifactorial resource limitations in LMICs. A recent study on pregnancy outcomes in women with heart disease from the Madras Medical College Pregnancy and Cardiac (M-PAC) registry describes the pregnancy outcomes in 70 women with a prosthetic heart valve from India.^8^ They found a very high foetal death rate (40%) and the occurrence of an adverse maternal cardiac outcome in 34%, including a high number of valve thromboses (17%). In a large cohort of 15 068 asymptomatic pregnant women not known to have heart disease in Pakistan who underwent antenatal echocardiographic screening, 4% of these women were found to have structural heart disease.^17^ This illustrates that there is an unmet clinical need for increased cardiovascular care in the pre-conception and early pregnancy periods. Our results complement those from these studies, reporting detailed information on anticoagulation and on monitoring and finding clear differences between HICs, where checks are more regular, and LMICs, where checks are less regular. It is of interest that, despite higher monitoring and better resource availability, the risk of thrombosis was higher in HICs, specifically due to higher use of LMWH. In addition, unsurprisingly, the cause of underlying valvular disease differed between the women from HICs and LMICs: in women from HICs, CHD was more often the cause of underlying valvular disease and rheumatic heart disease was more often the cause of underlying valvular disease in women from LMICs. Despite having limited data on the types of CHD, the complexity and multi-morbidity in women with CHD may also contribute to the higher incidence of adverse maternal cardiac outcomes during pregnancy.

### Strengths and limitations

Our study is the largest cohort of pregnant women with a prosthetic valve. All women were prospectively enrolled and already known to have a prosthetic valve before getting pregnant, which minimizes the risk of selection bias. Additionally, we have worldwide data which give an overview of global practice and data on anticoagulation levels at time of thromboembolic or haemorrhagic complications. Despite these strengths, our study has also several limitations. As with other registries, we have some missing data. However, the proportion of missing data is low as most of the variables are available in >95% of the pregnancies. Although TTE was performed and reported extensively, no specification is available on whether TEE was also performed and therefore the incidence of valve thrombosis may have been underestimated. On the other hand, in five women with valve thrombosis, no clear thrombus or mass was detected during echocardiogram alongside a new stenosis or immobile cusps, which could suggest an overestimation of the diagnosis. However, improvement of the valve function occurred in all these five women after heparin therapy or thrombolysis, which makes valve thrombosis highly likely. We have data on anticoagulation use in almost all pregnancies (99%); however, we have no data on dose adjustments during pregnancy. We gathered the peak anti-Xa target level for women using LMWH; however, it is unclear whether the anti-Xa levels reported at time of valve thrombosis represent also the peak levels or were measured at random. Also, it is not clear whether the high percentage of miscarriage and loss of pregnancy was anticoagulation related.

## Conclusions

Pregnancy in women with a prosthetic valve is high risk, with more favourable outcomes in women with a biological valve. We identified a mitral prosthetic valve and BMI as a predictor for valve thrombosis and any thromboembolic event, respectively, and these women should be followed even more careful with a low threshold to perform imaging including TEE. Anticoagulation regimens differ worldwide, probably due to differences in access to medical resources. We observed the highest risk of mechanical valve thrombosis while on LMWH. On the basis of our findings, we conclude that it is reasonable to maintain the status quo as contained in the current guidelines and continue to recommend anti-Xa monitoring of LMWH treatment if possible, on the basis that it is highly unlikely to be harmful, and there is a definite possibility that in some individual cases, it may be beneficial in avoiding anticoagulation levels falling too low or being excessive.

## Supplementary Material

ehaf265_Supplementary_Data

## Declarations

### Data Availability

The de-identified participant data that support the findings of this study are available from the corresponding author on reasonable request.

### Pre-registered Clinical Trial Number

None supplied.

## Appendix

**EORP Oversight Committee**

**2014–16**: R. Ferrari, IT (chair); A. Alonso, ES; J. Bax, NL; C. Blomström-Lundqvist, SE; S. Gielen, DE; P. Lancellotti, BE; A.P. Maggioni, IT; N. Maniadakis, GR; F. Pinto, PT; F. Ruschitzka, CH; L. Tavazzi, IT; P. Vardas, GR; F. Weidinger, AT; U. Zeymer, DE. **2016–18**: A. Vahanian, FR (chair); A. Budaj, PL; N. Dagres, DE; N. Danchin, FR; V. Delgado, NL; J. Emberson, GB; O. Friberg, SE; C.P. Gale, GB; G. Heyndrickx, BE; B. Iung, FR; S. James, SE; A.P. Kappetein, NL; A.P. Maggioni, IT; N. Maniadakis, GR; K.V. Nagy, HU; G. Parati, IT; A-S. Petronio, IT; M. Pietila, FI; E. Prescott, DK; F. Ruschitzka, CH; F. Van de Werf, BE; F. Weidinger, AT; U. Zeymer, DE. **2018–20**: C.P. Gale, GB (chair); B. Beleslin, RS; A. Budaj, PL; O. Chioncel, RO; N. Dagres, DE; N. Danchin, FR; J. Emberson, GB; D. Erlinge, SE; M. Glikson, IL; A. Gray, GB; M. Kayikcioglu, TR; A.P. Maggioni, IT; K.V. Nagy, HU; A. Nedoshivin, RU; A-P. Petronio, IT; J.W. Roos-Hesselink, NL; L. Wallentin, SE; U. Zeymer, DE. **2020–22**: B.A. Popescu, RO (chair); D. Adlam, GB; A.L.P. Caforio, IT; D. Capodanno, IT; M. Dweck, GB; D. Erlinge, SE; M. Glikson, IL; J. Hausleiter, DE; B. Iung, FR; M. Kayikcioglu, TR; P. Ludman, GB; L. Lund, SE; A.P. Maggioni, IT; S. Matskeplishvili, RU; B. Meder, DE; K.V. Nagy, HU; A. Nedoshivin, RU; D. Neglia, IT; A.A. Pasquet, BE; J.W. Roos-Hesselink, NL; F.J. Rossello, ES; S.M. Shaheen, EG; A. Torbica, IT.

**Executive Committee**

Jolien Roos-Hesselink, The Netherlands (co-chair); Roger Hall, UK (co-chair); William Parsonage, Australia; Werner Budts, Belgium; Julie de Backer, Belgium; Jasmin Grewal, Canada; Ariane Marelli, Canada; Guillaume Jondeau, France; Mark Johnson, UK; Catherine Otto, USA; Karen Sliwa, South Africa; Aldo P. Maggioni, Italy.

**Investigators**

**Armenia:**  *Yerevan:* K. Vardanyan, A. Melkonyan, H. Lachikyan, K. Hakobyan, M. Mazmanian; *Yerevan:* H. Hayrapetyan, A. Tavaracyan, H. Poghosyan, R. Hovhannisyan, S. Sahakyan, S. Martirosyan. **Australia:**  *St Leonards:* J. Harris. **Belgium:**  *Brussels:* A. Pasquet; *Brussels:* M. Morissens, T. Besse-Hammer, B. Dumoulin; *Gent:* J. De Backer, L. Campens, L. Demulier, M. De Hosson; *Leuven:* W. Budts, A. Van de Bruaene, A. Rampelberg, E. Troost, L. Roggen, P. De Meester. **Botswana:**  *Gaborone:* J.C. Mwita, E. Tefera, L. Kontle. **Canada:**  *Montreal:* A. Marelli, I. Malhamé; *Vancouver:* J. Grewal, M. Janzen. **Cuba:**  *La Habana:* P.A. Román Rubio, R. Vasallo Peraza, G. Vázquez Hernández, J.E. Pérez Torga, Y. Gil Jiménez, M. Meluzá Martín. **Egypt:**  *Cairo:* R. Almaleh, *Cairo:* G. Youssef, K. Sorour. **Ethiopia:**  *Addis Ababa:* S. Abebe, D. Mekonnen, C. Fekadu, D. Yadeta. **France:**  *Bron:* S. Dupuis-Girod, L. Delagrange; *Lille:* M. Richardson, L. Ghesquiere, O. Domanski, M. Gonzalez Estevez, Y. Ould Hamoud, S. Gautier, L. Marsili; *Marseille:* L. Bal-Theoleyre, S. Palazzolo; *Paris:* M. Ladouceur; *Paris:* G. Jondeau, A. Bourgeois Moine, L. Eliaou, O. Milleron, M. Tchitchinadze; *Toulouse:* Y. Dulac, C. Karsenty, N. Souletie, F. Bajanca. **Germany:**  *Hamburg:* C. Rickers, S. Blankenberg, C. Sinning, C. Magnussen, E. Zengin, G. Mueller, R. Schnabel, Y. Von Kodolitsch, R. Kozlik-Feldmann; *Muenster:* H. Baumgartner, R. Schmidt, A. Hellige, A. Rietkötter. **Greece:**  *Athens:* M. Spartalis; *Athens:* A.A. Frogoudaki; *Thessaloniki:* A. Arvanitaki, A. Baroutidou, G. Giannakoulas, C. Karvounis. **India:**  *Chennai:* J.P. Gnanaraj, A.P. Steaphen, T. Ethirajan, K. Kannan, V. Subramanian, A. Surendran, J. Gnanasekaran, V. Natarajalingam, S. Balasubramani. **Iraq:**  *Bagdad:* H. Ali Farhan, I.F. Yaseen. **Italy:**  *Bologna:* E. Mariucci, C. Ciuca; *Massa:* F. Marchi, G. Benedetti, M. Baroni, P. Festa, A. Parlanti; *Naples:* G. Scognamiglio, F. Fusco, B. Sarubbi; *Trieste:* M. Merlo, B. D'Agata Mottolese, C. Carriere, G. Sinagra, M. Bobbo, F. Ramani; *Turin:* F.M. Comoglio, R. Bordese, A. Pagano, N. Montali, V. Donvito, C.A. Remolif, F. Petey. **Netherlands:**  *Amsterdam:* B. Bouma, S. Chamuleau, D. Robbers-Visser; *Amsterdam:* T. Konings, H. Dronkert; *Breda:* D. Segers; *Nijmegen:* R. Van Kimmenade; *Utrecht:* H. Van Der Zwaan, G. Tjalling Sieswerda, A. Evers, T. Schaap. **Pakistan:**  *Karachi:* K. Bano, H. Yasmeen, K. Amir, N. Patel, P. Akhter, R. Khan, A. Shakeel, S. Mahar, S. Habib. **Poland:**  *Lodz:* M. Lelonek; *Warsaw:* P. Hoffman, M. Lipczynska. **Portugal:**  *Lisbon:* L. De Sousa, V. Ferreira, T. Mano, M. Selas, R. Cruz Ferreira. **Russian Federation:**  *Saint Petersburg:* E. Shlyakhto, O. Irtyuga, G. Sefieva, K. Malikov, T. Pervunina, U. Shadrina. **Saudi Arabia:**  *Jeddah:* A. Kinsara; *Riyadh:* D. Galzerano, H. Al Sergani, W. Kurdi, N. Kholaif, O. Vriz, A. Alhamshari, A. Alsaigh, O. Ahmad, S. Alzaher, B. Alamro. **South Africa:**  *Cape Town:* K. Sliwa, F. Jakoet-Bassier. **Spain:**  *Barcelona:* L. Galian-Gay, A. Pijuan-Domenech, B. Miranda-Barrio, B. Gordon. **Sweden:**  *Goteborg:* E. Furenäs; *Lund:* J. Hlebowicz, F. Wedlund; *Stockholm:* E. Nagy, E. Mattsson, M. Majczuk Sennstrom, P. Sörensson; *Uppsala:* C. Christersson, A. Lutvica, B. Jönelid, T. Achter, K. Junus, H. Gärdesten Wall, M. Andreasson. **Switzerland:**  *Basel:* D. Tobler; *Genève:* J. Bouchardy, F. Brand, C. Blanche; *Lausanne:* J. Bouchardy, T. Rutz, F. Brand. **Türkiye:**  *Ankara:* U. Canpolat, Y.Z. Sener, N. Ozer; *Istanbul:* E. Ayduk Gövdeli, Z. Bugra, B. Umman, P. Karaca Özer, D. Baykiz; *Istanbul:* D. Mutlu, H. Yalman, B. Kilickiran Avci; *Istanbul/Turkey:* S. Catirli Enar, O. Batukan Esen, D. Oksen. **Ukraine:**  *Kyiv:* V. Lazoryshynets, S. Siromakha, Y. Davydova, A. Limanska, I. Zinovchyk, V. Kravchenko, B. Kravchuk, O. Kravets, N. Volkova, O. Mazur, O. Beregovyi. **United Arab Emirates:**  *Abu Dhabi:* B.T. Salih, W.A.R. Al Mahmeed, S. Wani, F.S. Mohamed Farook, G. Al Mansoori. **USA:**  *Houston:* S. Prakash, R. Afifi, D. Milewicz, A. Cecchi; *Lexington, KY:* G. Wells, D. Sparks; *Minneapolis, MN:* W. Wagner, C. Bigelow, L. Colicchia, T. Jentink, M. Loichinger, R. Saxena, W. Wunderlich, C. Longtin, P. Klopper, R. Gobar; *New Haven:* J. Chou, K. Campbell, R. Elder; *New York:* D. Halpern, A. Hausvater, H. Reynolds, N. Bhalla, A. Small, J. Feinberg, P. Panday; *Phoenix:* J. Awerbach, J. Porche, S. Stack; *Portland:* L. Mcgrath, A. Khan, E. Pare, P. Woods, C. Broberg, K. Gibbins, K. Brookfield; *Stony Brook:* M. Al-Sadawi, A. Cove, N. Mann.
