# Supplementary material for: Pregnancy with a prosthetic heart valve, thrombosis, and bleeding: the ESC EORP Registry of Pregnancy and Cardiac disease III
Source: Eur Heart J. 2025 Apr 16;47(11):1318–35. doi: 10.1093/eurheartj/ehaf265 (PMC13016632; doi:10.1093/eurheartj/ehaf265)
Supplement: ehaf265_Supplementary_Data [file ehaf265_supplementary_data.docx]

**Supplementary Material**

**Supplementary Table 1.** Pre-pregnancy baseline characteristics and definitions of pregnancy outcomes.

Pre-pregnancy baseline characteristics were recorded, including demographics, cardiac diagnosis, prior interventions, medication use, obstetric history, initial assessment including heart rhythm and echocardiography, and whether pre-conception counselling was performed. The New York Heart Association (NYHA) functional classification was used to categorize patients according to their limitations of physical activity. The following data relating to the prosthetic valve were collected: underlying valvular disease, time since valve replacement, type and position of valve prosthesis, and prosthetic valve complications. Furthermore, cardiac and obstetric events during pregnancy, including interventions, were documented. Cardiac events up to one-week postpartum included maternal mortality, hospitalization, heart failure, thromboembolic events, hemorrhagic events, endocarditis, aortic dissection, arrhythmia and ischemic cardiac events. Adverse maternal cardiac outcome was defined as the combined endpoint of maternal mortality, heart failure, thromboembolic event, hemorrhagic event (including obstetric hemorrhages; any bleeding leading to a decline of Hb>1.0 point or any bleeding with severe consequences), endocarditis and (clinically relevant) arrhythmia, including new atrial fibrillation/flutter or ventricular tachycardia (≥3 consecutive ventricular beats with mean rate >100 beats per minute). Obstetric events were pregnancy-induced hypertension (PIH; new hypertension, systolic blood pressure >140 mmHg or diastolic blood pressure >90 mmHg, without end-organ findings, occurring after ≥20 weeks of gestation), pre-eclampsia (PIH with end-organ findings, including proteinuria), eclampsia or hemolysis, elevated liver enzymes and lower platelets (HELLP) syndrome (pre-eclampsia with grand mal seizures/HELLP syndrome) and gestational diabetes (diagnosed according to local criteria). Hypertensive disorder of pregnancy was defined as composite outcome of PIH, pre-eclampsia and eclampsia/HELLP syndrome. Fetal outcomes consisted of early miscarriage (fetal death <14 weeks of gestation), late miscarriage (fetal death 14-24 weeks of gestation), stillbirth (fetal death ≥24 weeks of gestation), termination of pregnancy, and fetal growth restriction. Delivery outcomes (mode of delivery, maternal cardiac and obstetric complications) were recorded if a delivery occurred ≥16 weeks of gestation, regardless of late miscarriage or stillbirth. Emergency Cesarean section was defined as taking place if the time interval between decision to deliver due to obstetric or cardiac indications and the Cesarean section was <24 hours. Postpartum hemorrhage was defined as hemorrhage until one week postpartum. Neonatal outcomes were recorded if a delivery and no fetal death occurred and included gestational age at birth, sex, birth weight, small for gestational age (birth weight <10^th^ percentile), congenital disease and neonatal mortality. Serious adverse events were maternal mortality, thromboembolic event, major hemorrhage, arrhythmia, heart failure, endocarditis, pre-eclampsia, HELLP and fetal loss. An uncomplicated pregnancy with a live birth was defined as a pregnancy without any of these serious adverse events.

Women with ≥2 pregnancies included in the ROPAC III were identified by comparing the month and year or birth, as well as the center where the data was entered, the data of previous interventions, and the data entered for earlier pregnancies, such as year of pregnancy and complications.

**Supplementary Table 2.** Description of the statistical analyses.

Statistical analyses were performed using IBM SPSS Statistics version 25.0. Categorical data were presented as numbers with percentages and continuous data as means with standard deviation or median with interquartile range if skewed (tested with one-sample Kolmogorov-Smirnov tests). Differences in categorical data were calculated by chi-quadrate tests or Fisher’s exact tests (if any expected cell count was <5). Differences in continuous data were calculated by Student’s t-tests, one-way analysis of variance, or Mann-Whitney U tests, as appropriate. P-values <0.05 (two-sided test) were considered statistically significant. Pre-pregnancy baseline characteristics and pregnancy outcomes were compared between women with a mechanical valve and women with a biological valve. Women with both a mechanical and biological valve were included in the mechanical valve group.

An univariate and multivariate logistic regression analysis was performed to explore for associations between an adverse maternal outcomes and pre-pregnancy baseline characteristics.

We performed an univariate logistic regression analysis to identify predictors for valve thrombosis, and subsequently, we performed a multivariate analysis including all variables that were p<0.1 in the univariate analysis and the position of the mechanical valve (mitral or aortic position). Also, we performed an univariate and multivariate logistic regression analysis to explore predictors for all thromboembolic events in women with a mechanical valve, including all variables with p<0.1 in the univariate analysis. Additionally, baseline characteristics and pregnancy outcomes were compared between LMIC and HIC, based on the International Monetary Fund Classification (7), and between the different anticoagulation regimes used during pregnancy using Bonferroni correction for multiple testing. Lastly, VKA average daily dosages within the women with a mechanical valve who used VKA throughout pregnancy were divided into quartiles of warfarin equivalent doses (<4mg, 4.0-4.9mg, 5.0-5.9mg, ≥6mg) and fetal and neonatal outcomes were reported. The average daily doses of VKAs other than warfarin (acenocoumarol and fenprocoumon) were converted to their warfarin equivalent doses, with a conversion factor of 1:1.85 and 1:2.36, respectively (x mg drug : y mg warfarin) (8).

*7. World Economic Outlook (WEO). Growth Resuming, Dangers Remain. 2012.*

*8. van Leeuwen Y, Rosendaal FR, van der Meer FJ. The relationship between maintenance dosages of three vitamin K antagonists: acenocoumarol, warfarin and phenprocoumon. Thromb Res. 2008;123(2):225-30.*

| **Supplementary Table 3**. Missing data in ROPAC III | |
| --- | --- |
|  | **Missing - N (%)** |
| **Pre-pregnancy baseline characteristics** |  |
| Age | 80 (13.1) |
| BMI | 13 (2.1) |
| Nulliparity | 4 (0.7) |
| LMIC | 9 (2.2) |
| Current smoker | 1 (0.2) |
| Chronic hypertension | 1 (0.2) |
| Atrial fibrillation / flutter | 14 (2.3) |
| Diabetes mellitus | 1 (0.2) |
| Chronic kidney disease | 2 (0.3) |
| Clinical signs of heart failure | 5 (0.8) |
| Estimated SEF <40% | 22 (3.6) |
| NYHA class >II | 24 (3.9) |
| Cyanosis | 11 (1.8) |
| Non-cardiac disease | 4 (0.7) |
| Anticoagulation use | 5 (0.8) |
| Platelet aggregation inhibitor use | 5 (0.8) |
| Cardiac medication use | 5 (0.8) |
|  |  |
| **Prosthetic valve details** |  |
| Position | - |
| Age at first valve replacement | 98 (16.0) |
| Time first valve replacement to current pregnancy | 102 (16.6) |
| Cause of underlying valvular disease | - |
| Malfunction before pregnancy | 77 (12.6) |
|  |  |
| **Maternal outcomes** |  |
| Adverse maternal cardiac outcome | 27 (4.4) |
| Maternal mortality | 11 (1.8) |
| Heart failure | 27 (4.4) |
| Thromboembolic event | 17 (2.8) |
| Hemorrhagic event | 21 (5.1) |
| Endocarditis | 18 (2.9) |
| Arrhythmia | 19 (3.1) |
| Hypertensive disorder | 7 (1.1) |
| Gestational diabetes | 8 (1.3) |
|  |  |
| **Fetal outcomes** |  |
| Fetal death | 12 (1.9) |
| Therapeutic abortion | 15 (2.4) |
| Intra-uterine growth restriction | 10 (1.6) |
|  |  |
| **Mode of delivery** |  |
| Cesarean section | 12 (2.3) |
| Emergency cesarean section | 16 (3.0) |
| Reason for emergency cesarean section | - |
|  |  |
| **Neonatal outcomes** |  |
| Gestational age at delivery | 11 (2.3) |
| Preterm birth | 11 (2.3) |
| Birth weight | 37 (7.7) |
| Small for gestational age | 37 (7.7) |
| Congenital heart disease | 29 (6.0) |
| Other congenital disease | 28 (5.8) |
| Neonatal mortality | 1 (0.2) |
|  |  |
| **Anticoagulation regimen** |  |
| Anticoagulation regimen | 3 (0.7) |

Data are presented as n (%) and relate to the number of pregnancies.

Abbreviations: BMI, body mass index; LMIC, low-or-middle-income country; NYHA, New York Heart Association classification; SEF, systemic ejection fraction.

| **Supplementary Table 4**. Position of the prosthetic valves in the total cohort and stratified by type of valve | | | | |
| --- | --- | --- | --- | --- |
|  | Total cohort (n=613) | Mechanical valve (n=411)^a^ | Biological valve (n=202) | P-value^b^ |
| **Position of the prosthetic valve(s)** |  |  |  |  |
| Aortic | 158 (25.8) | 122 (29.7) | 36 (17.8) | **<0.001** |
| Mitral | 263 (42.9) | 225 (54.7) | 38 (18.8) | **<0.001** |
| Pulmonary | 83 (13.5) | 1 (0.2) | 82 (40.6) | **<0.001** |
| Tricuspid | 14 (2.3) | 3 (0.7) | 11 (5.4) | **<0.001** |
| Aortic + Mitral | 62 (10.1) | 50 (12.2) | 12 (5.9) | **0.022** |
| Aortic + Pulmonary^c^ | 27 (4.4) | 6 (1.5) | 21 (10.4) | **<0.001** |
| Aortic + Tricuspid | 1 (0.2) | 0 (0) | 1 (0.5) | 0.330 |
| Mitral + Tricuspid | 2 (0.3) | 1 (0.2) | 1 (0.5) | 0.551 |
| Aortic + Mitral + Tricuspid | 2 (0.3) | 2 (0.5) | 0 (0) | 1.000 |
| Aortic + Pulmonary + Tricuspid | 1 (0.2) | 1 (0.2) | 0 (0) | 1.000 |

Data are presented as n (%) unless otherwise specified, and relate to the number of pregnancies.

^a^Nine pregnancies were in women with both a mechanical and biological valve during current pregnancy; included in mechanical valve group.

^b^P-value for mechanical valve versus biological valve.

^c^Ross procedure in 19 (70%) out of 27 pregnancies in women with an aortic and pulmonary prosthetic valve.

**Supplementary Table 5.** Univariate and multivariate logistic regression analysis for an adverse maternal cardiac outcome during pregnancy in women with a prosthetic valve.

|  | **Univariate** | | | **Multivariate** | | |
| --- | --- | --- | --- | --- | --- | --- |
|  | Odds ratio | 95% CI  Lower-Upper limit | p-value | Odds ratio | 95% CI  Lower-Upper limit | p-value |
| Age | 1.01 | 0.97-1.04 | 0.735 | 0.99 | 0.95-1.03 | 0.536 |
| BMI | 1.03 | 1.00-1.06 | 0.082 | 1.03 | 0.99-1.07 | 0.137 |
| Nulliparity | 1.00 | 0.68-1.49 | 0.990 | 0.87 | 0.54-1.39 | 0.547 |
| LMIC | 0.58 | 0.40-0.86 | **0.006** | 0.42 | 0.25-0.72 | **0.002** |
| Current smoker | 1.67 | 0.56-4.96 | 0.357 | 0.98 | 0.26-3.76 | 0.978 |
| Chronic hypertension | 1.67 | 0.69-4.02 | 0.255 | 1.15 | 0.39-3.41 | 0.806 |
| Diabetes mellitus | 3.96 | 0.66-23.82 | 0.133 | 2.13 | 0.21-22.19 | 0.527 |
| Atrial fibrillation/flutter | 8.91 | 3.27-24.27 | **<0.001** | 12.69 | 4.16-38.80 | **<0.001** |
| Signs of heart failure | 4.08 | 2.00-8.34 | **<0.001** | 3.09 | 1.23-7.73 | **0.016** |
| SEF <40% | 3.57 | 1.17-10.93 | **0.026** | 2.38 | 0.65-8.69 | 0.190 |
| NYHA class >II | 4.85 | 1.64-14.38 | **0.004** | 3.00 | 0.75-11.92 | 0.119 |
| Non-cardiac disease | 2.33 | 1.27-4.27 | **0.006** | 1.36 | 0.66-2.80 | 0.410 |
| Cardiac medication before pregnancy | 1.47 | 0.81-2.66 | 0.204 | 1.41 | 0.71-2.83 | 0.328 |
| Mechanical valve | 1.62 | 1.06-2.47 | **0.025** | 3.23 | 1.83-5.68 | **<0.001** |
| Congenital heart disease | 1.25 | 0.85-1.82 | 0.256 | 1.73 | 0.89-3.37 | 0.109 |
| Rheumatic heart disease | 0.74 | 0.50-1.08 | 0.118 | 1.00 | 0.51-1.99 | 0.992 |
| Malfunction of prosthetic valve before pregnancy | 1.26 | 0.70-2.27 | 0.439 | 1.47 | 0.72-2.97 | 0.288 |

Abbreviations: BMI, body mass index; LMIC, low-or-middle income country; NYHA, New York Heart Association; SEF, systemic ejection fraction.

Logistic regression not possible for chronic kidney disease due to quasi separation.

**Supplementary Table 6**. Maternal mortality cases, details

|  | Maternal age, years | GxPx | LMIC | Cardiac diagnosis | Comorbidity | NYHA class | Time of death | Cause of death |
| --- | --- | --- | --- | --- | --- | --- | --- | --- |
| 1. | 27 | G2P0 | Yes | CHD, MVP | None | I | During pregnancy (AD 10 weeks) | Valve thrombosis, heart failure |
| 2. | Unknown | G4P3 | Yes | RHD, MVP | None | II | After delivery | Rapid AF during delivery, high gradients across MVP, cardiogenic shock |
| 3. | Unknown | G3P1^a^ | Yes | CHD, MVP + AVP | None | II | During pregnancy (AD 7 weeks) | Valve thrombosis, heart failure |
| 4. | 31 | G2P1 | Yes | CHD, MVP | Factor V Leiden | I | During pregnancy (AD 13 weeks) | Sudden cardiac death at home |

AD, amenorrhea duration; AF, atrial fibrillation; AVP, aortic valve prosthesis; CHD, congenital heart disease; GxPx, Gravida Para; LMIC, low-or-middle-income country; MVP, mitral valve prosthesis; NYHA, New York Heart Association classification; RHD, rheumatic heart disease.

^a^Intra-uterine death.

**Supplementary Table 7**. Details on valve thrombosis cases in the ROPAC III

|  | LMIC | Maternal age, years | Cardiac diagnosis | Valve position | GxPx, Complications previous pregnancies | Time between VR and current pregnancy, years | Gestational age during MVT, weeks | Findings on echocardiogram^d^ | Anticoagulation during MVT | Anticoagulation recently switched (<2 weeks) | Anti-Xa or INR target level | Anti-Xa, INR, PTT during MVT | Treatment^c^ | Fetal outcome |
| --- | --- | --- | --- | --- | --- | --- | --- | --- | --- | --- | --- | --- | --- | --- |
| 1. | No | 34 | CHD | Aortic | G3P0  Two miscarriages | 23 | 13 | Mean PG 59mmHg | LMWH  Aspirin (80mg) | No | Anti-Xa not measured  INR 2.0-3.0 | - | IV heparin | Good |
| 2. | No | 39 | RHD | Mitral | G1P0 | 1 | 30 | Large mass on prosthesis | LMWH  Aspirin (80mg) | No | Anti-Xa 1.0-1.2  INR 2.5-3.5 | Anti-Xa 0.9  INR 0.97  PTT 61s | Valve replacement (biological) | Preterm birth at 30 weeks |
| 3. | No | 32 | RHD | Mitral | G1P0 | 10 | 31 | Immobile leaflet  Mean PG 29mmHg | LMWH | No | Anti-Xa 0.6-1.0  INR 2.5-3.5 | Anti-Xa 0.5  INR 1.0 | Thrombolysis | Preterm birth at 31 weeks |
| 4.^a^ | Yes | 27 | CHD | Mitral | G2P0  Valve thrombosis, therapeutic abortion | 13 | 10 | Thrombus | UFH | No | INR target level unknown | INR 1.4  PTT 68s | IV heparin > Thrombolysis | Fetal demise at 10 weeks |
| 5. | No | 34 | RHD | Mitral | G1P0 | 18 | 27 | Thrombus | LMWH | No | Anti-Xa 1.0-1.2 | Anti-Xa 0.9  INR 1.0  PTT 29s | IV heparin | Preterm birth at 28 weeks |
| 6.^a^ | Yes | - | Other | Aortic + Mitral^b^ | G3P1  One miscarriage, one IUFD | - | 7 | Stuck leaflet, Mean PG 24mmHg | UFH | No | INR 2.5-3.5 | PTT 50s | Valve replacement (mechanical) | Fetal demise at 7 weeks |
| 7. | No | 35 | RHD | Mitral | G1P0 | 6 | 14 | Multiple thrombi | LMWH | No | Anti-Xa not measured | - | Valve replacement (mechanical) | SGA |
| 8. | No | 33 | Endocarditis | Mitral | G1P0 | 3 | 11 | Thrombus  Mean PG 17mmHg | LMWH | No | Anti-Xa not measured | - | IV heparin > valve replacement (mechanical) | Twin pregnancy Preterm birth at 36 weeks |
| 9. | No | 39 | RHD | Aortic + Mitral^b^ | G6P2  Three miscarriage, child with Binder syndrome | 17 | 15 | Thrombus  Mean PG 20mmHg | LMWH  Aspirin (75mg) | No | Anti-Xa 0.6-1.0 | Anti-Xa 0.9 | IV heparin > valve replacement (mechanical) | Good |
| 10. | Yes | 30 | RHD | Mitral | G2P1  No complications | 4 | 31 | New stenosis  Mean PG 23mmHg | LMWH | No | Anti-Xa not measured | INR 0.8  PTT 29s | IV heparin | IUFD at 31 weeks |
| 11. | Yes | 27 | Unknown | Aortic | G6P5  No complications | 1 | 22 | Echodense mass Mean PG 47mmHg | VKA | No | INR target level unknown | INR 1.2 | IV heparin > thrombolysis | IUFD at 28 weeks |
| 12. | Yes | - | Unknown | Aortic | G1P0 | - | 14 | Thrombus  Mean PG 42mmHg | VKA | Yes  (LMWH>VKA)  1 week prior | Anti-Xa not measured  INR target level unknown | INR 2.1 | IV heparin > thrombolysis | Unknown |
| 13. | No | 21 | Other | Aortic | G1P0 | 6 | 30 | New stenosis  Mean PG 75mmHg | LMWH | No | Anti-Xa 0.6-1.0 | Anti-Xa unknown | Valve replacement (mechanical) | Preterm birth at 30 weeks |
| 14. | No | 22 | CHD | Mitral | G3P0  One miscarriage | 10 | 8 | New moderate stenosis | LMWH | No | Anti-Xa target level unknown  INR 2.5-3.5 | Anti-Xa unknown  INR 1.0  PTT 29s | Thrombolysis | Good |
| 15. | No | 33 | CHD | Mitral | G2P0  Valve thrombosis, miscarriage | 17 | 10 | Thrombus  Mean PG 28mmHg | LMWH | No | Anti-Xa 1.0-1.2  INR 2.5-3.5 | Anti-Xa 0.6 | Thrombolysis | Good |
| 16. | No | 36 | CHD | Aortic^b^ + Pulmonary | G2P0  Miscarriage | 18 | 13 | Thrombus | LMWH | No | Anti-Xa 1.0-1.2  INR 2.0-3.0 | Anti-Xa 0.9 | IV heparin > thrombolysis | Preterm birth at 36 weeks |
| 17. | Yes | 37 | RHD | Mitral | G4P3  No complications | - | 24 | Unknown | VKA | No | INR 2.5-3.5 | INR 1.2 | IV heparin | IUFD at 25 weeks |
| 18. | Yes | 41 | CHD | Mitral | G4P3  Unknown | 5 | 25 | Immobile cusps | LMWH | No | Anti-Xa 0.6-1.0  INR 2.5-3.5 | INR 1.1  PTT 40s | IV heparin | Good |
| 19. | Yes | 37 | Other | Mitral | G3P2  No complications | 7 | 24 | Echodense mass on leaflet | LMWH | No | Anti-Xa 1.0-1.2 | Anti-Xa 0.7 | IV heparin | Preterm birth at 32 weeks, SGA |
| 20. | Yes | 40 | Endocarditis | Mitral | G10P6  Three miscarriages | 11 | 13 | Severe stenosis | VKA | No | INR target level unknown | INR 2.0  PTT 52s | Valve replacement (biological) | Fetal death at 13 weeks |
| 21. | Yes | 40 | RHD | Aortic + Mitral^b^ | G1P0 | 7 | 16 | Mobile thrombus  Mean PG 16mmHg | LMWH | No | Anti-Xa 1.0-1.2  INR 2.5-3.5 | Anti-Xa 0.6  INR 1.1  PTT 51s | Valve replacement after termination | Termination of pregnancy due to maternal condition at 18 weeks |
| 22. | Yes | 28 | Degenerative | Mitral | G1P0 | 6 | 39 | Large thrombus (1.2cm) | LMWH | No | Anti-Xa 1.0-1.2  INR 2.0-3.0 | Anti-Xa 1.2  PTT 37s | Thrombectomy | Good |
| 23. | No | 36 | RHD | Mitral | G1P0 | 15 | 25 | Large mobile thrombus | LMWH | No | Anti-Xa not measured  INR 2.5-3.5 | INR 1.0  PTT 34s | Valve replacement (mechanical) | Preterm birth at 27 weeks |
| 24. | Yes | 26 | RHD | Mitral | G3P2  One IUFD | - | 33 | Mobile thrombus | VKA | No | INR 2.5-3.5 | INR 1.2 | IV heparin | SGA |

Abbreviations: AVSD, atrioventricular septal defect; CHD, congenital heart disease; CS, cesarean section; GxPx, Gravida Para; INR, International normalized ratio; IUFD, intra-uterine fetal death; LMIC, low-or-middle-income country; LMWH, low-molecular weight heparin; MVT, mechanical valve thrombosis; PG, peak gradient; PTT, partial thromboplastin time; RHD, rheumatic heart disease; SGA, small for gestational age; UFH, unfractionated heparin; VKA, vitamin K antagonist; VR, valve replacement.

^a^Maternal mortality due to valve thrombosis.

^b^Indicates in which valve the thrombosis occurred in case of double mechanical valve.

^c^Valve replacement or thrombectomy in all cases after cesarean section unless otherwise specified.

^d^In case 6, 13 and 30, the diagnosis valve thrombosis was confirmed during valve replacement. In case 1, 3, 10, 14 and 18, improvement of valve function occurred after IV heparin therapy or thrombolysis.

**Supplementary Table 8**. Details on other thromboembolic events in women with a mechanical valve.

|  | LMIC | Maternal age, years | Cardiac diagnosis | Valve position | GxPx, Complications previous pregnancies | Type of TE | Gestational age during TE, weeks | Anticoagulation during TE | Anticoagulation recently switched (<2 weeks) | Anti-Xa, INR, PTT during TE | Treatment | Fetal outcome |
| --- | --- | --- | --- | --- | --- | --- | --- | --- | --- | --- | --- | --- |
| 1. | Yes | 29 | CHD | Mitral | G1P0 | Stroke | 8 | UFH | Unknown | INR 1.2  PTT 65s | IV UFH | Therapeutic abortion |
| 2. | Yes | 24 | CHD | Mitral | G1P0 | Stroke | 19 | VKA | Unknown | INR 2.8 | IV UFH | Fetal death at 19 weeks |
| 3. | Yes | 24 | RHD | Aortic + Mitral | G3P0  Miscarriage, IUFD | Stroke | 6 | Unknown | Unknown | Unknown | Unknown | Good |
| 4. | Yes | 31 | RHD | Aortic | G3P1  IUFD | Stroke | 30 | LMWH | Unknown | INR 1.2 | Unknown | Good |
| 5. | Yes | 30 | RHD | Mitral | G3P1  Miscarriage | TIA | 5 | UFH | No | PTT 35s | Unknown | SGA |
| 6. | Yes | 27 | RHD | Mitral | G3P0  Miscarriage,  IUFD | TIA | 20 | LMWH | Unknown | Unknown | Unknown | SGA |
| 7. | Yes | - | RHD | Mitral | G3P1  Miscarriage,  MVR^a^ | Stroke | 29 | Unknown | Unknown | Unknown | Unknown | Good |
| 8. | Yes | - | CHD | Mitral | G4P3 | Unknown | 34 | LMWH | Yes | Unknown | IV UFH | Neonatal death due to hydrocephaly |
| 9. | No | 42 | CHD | Aortic + Pulmonary | G7P6 | TIA | 10 | LMWH | No | INR 1.0  Anti-Xa 0.6 | LMWH | Good |
| 10. | Yes | 23 | CHD^b^ | Aortic | G2P1 | Stroke + MI | 9 | LMWH | Unknown | INR 1.2  Anti-Xa 0.2  PTT 28s | LMWH + PCI | Preterm birth at 36 weeks |

Abbreviations: CHD, congenital heart disease; GxPx, Gravida Para; INR, International normalized ratio; IUFD, intra-uterine fetal death; LMIC, low-or-middle-income country; LMWH, low-molecular weight heparin; MI; myocardial infarction; MVT, mechanical valve thrombosis; PCI, percutaneous coronary intervention; PTT, partial thromboplastin time; RHD, rheumatic heart disease; SGA, small for gestational age; UFH, unfractionated heparin; VKA, vitamin K antagonist; MVR, mitral valve replacement.

^a^Redo mitral valve replacement during previous pregnancy due to an acute stuck mitral valve prosthesis.

^b^Also known with genetic thrombophilia, antithrombin III deficiency.

**Supplementary Table 9.** Univariate and multivariate logistic regression analysis for any thromboembolic event during pregnancy in women with a mechanical valve.

|  | **Univariate** | | | **Multivariate** | | |
| --- | --- | --- | --- | --- | --- | --- |
|  | Odds ratio | 95% CI  Lower-Upper limit | p-value | Odds ratio | 95% CI  Lower-Upper limit | p-value |
| Age | 1.05 | 0.99-1.12 | 0.134 |  |  |  |
| BMI | 1.07 | 1.01-1.13 | **0.019** | 1.07 | 1.01-1.13 | **0.020** |
| Nulliparity | 1.00 | 0.48-2.09 | 0.997 |  |  |  |
| LMIC | 0.36 | 0.17-0.75 | **0.007** | 0.36 | 0.17-0.75 | **0.007** |
| Current smoker | 1.74 | 0.20-14.97 | 0.611 |  |  |  |
| Chronic hypertension | 0.62 | 0.08-4.78 | 0.644 |  |  |  |
| Atrial fibrillation/flutter | 2.84 | 0.75-10.66 | 0.123 |  |  |  |
| Signs of heart failure | 1.29 | 0.67-4.49 | 0.695 |  |  |  |
| SEF <40% | 0.99 | 0.12-7.93 | 0.992 |  |  |  |
| Non-cardiac disease | 3.00 | 1.13-7.95 | **0.027** | 1.86 | 0.63-5.53 | 0.264 |
| Cardiac medication before pregnancy | 0.32 | 0.04-2.41 | 0.267 |  |  |  |
| Congenital heart disease | 1.39 | 0.65-2.96 | 0.395 |  |  |  |
| Rheumatic heart disease | 0.60 | 0.29-1.21 | 0.153 |  |  |  |
| Mitral position | 1.58 | 0.76-3.29 | 0.222 |  |  |  |
| Aortic position | 0.49 | 0.20-1.21 | 0.121 |  |  |  |
| Malfunction of prosthetic valve before pregnancy | 1.02 | 0.13-7.87 | 0.989 |  |  |  |
| Plan for anticoagulation monitoring | 1.00 | 0.99-1.02 | 0.787 |  |  |  |

Abbreviations: BMI, body mass index; LMIC, low-or-middle-income country; SEF, systemic ejection fraction.

Logistic regression not possible for Diabetes mellitus, renal disease and NYHA class >II due to quasi separation.

| **Supplementary Table 10.** Maternal outcomes excluding women with two or more pregnancies in ROPAC | | | | |
| --- | --- | --- | --- | --- |
|  | Total  (n=560) | Mechanical valve  (n=385) | Biological valve  (n=175) | P-value |
| **Adverse maternal cardiac outcome** | 127 (23.9) | 97 (26.7) | 30 (17.9) | **0.029** |
| Maternal mortality | 4 (0.7) | 4 (1.1) | 0 (0) | 0.313 |
| Heart failure | 33 (5.9) | 22 (5.7) | 11 (6.3) | 0.847 |
| Thromboembolic event | 36 (6.6) | 33 (8.9) | 3 (1.7) | **0.003** |
| Valve thrombosis | 23 (4.1) | 23 (6.0) | 0 (0) | **<0.001** |
| Hemorrhagic event | 79 (14.8) | 66 (18.1) | 13 (7.7) | **0.002** |
| Arrhythmia | 19 (3.6) | 13 (3.6) | 6 (3.6) | 1.000 |
| Endocarditis | 3 (0.6) | 3 (0.8) | 0 (0) | 0.555 |

Data are presented as n (%) and relate to number of pregnancies.

^a^Twenty-five women are excluded from this analysis: 23 with two, one with three, and one woman with four included pregnancies. Twelve out of 25 women had a mechanical valve, of which one was diagnosed with valve thrombosis during her second pregnancy.

**Supplementary Table 11.** Pre-pregnancy baseline characteristics and pregnancy outcomes of pregnancies in women with a mechanical valve, stratified by different anticoagulation regimens

|  | Total (n=408) | Regimen 1^a^  (n=120) | Regimen 2^b^  (n=72) | Regimen 3^c^  (n=137) | Regimen 4^d^  (n=79) | P-value* |
| --- | --- | --- | --- | --- | --- | --- |
| **Baseline characteristics** |  |  |  |  |  |  |
| Age, years, mean ± SD | 29.8 ± 5.9 | 29.2 ± 5.9 | 31.7 ± 5.9 | 27.9 ± 5.6 | 31.3 ± 5.6 | **<0.001** |
| BMI, kg/m^2^, median (Q1-Q3) | 24.9 (22.5-28.1) | 25.8 (22.7-28.0) | 24.8 (22.5-29.4) | 24.6 (22.7-28.4) | 24.7 (22.1-27.8) | 0.589 |
| Nulliparity | 140 (34.3) | 35 (29.2) | 26 (36.1) | 42 (30.7) | 37 (46.8) | 0.049 |
| LMIC | 331 (81.1) | 109 (90.8) | 47 (65.3) | 136 (99.3) | 39 (49.4) | **<0.001** |
| Current smoker | 7 (1.7) | 1 (0.8) | 2 (2.8) | 3 (2.2) | 1 (1.3) | 0.799 |
| Chronic hypertension | 18 (4.4) | 5 (4.2) | 2 (2.8) | 2 (1.5) | 9 (11.4) | 0.011 |
| Diabetes mellitus | 3 (0.7) | 1 (0.8) | 1 (1.4) | 0 (0) | 1 (1.3) | 0.457 |
| Renal disease | 1 (0.2) | 0 (0) | 0 (0) | 0 (0) | 1 (1.3) | 0.367 |
| Clinical signs of heart failure | 33 (8.2) | 16 (13.3) | 4 (5.6) | 5 (3.7) | 8 (10.4) | 0.028 |
| Estimated SEF <40% | 12 (3.1) | 4 (3.6) | 3 (4.2) | 4 (3.1) | 1 (1.4) | 0.781 |
| NYHA class >II | 14 (3.6) | 6 (5.2) | 2 (3.1) | 3 (2.2) | 3 (3.9) | 0.636 |
| Non-cardiac disease | 32 (7.9) | 2 (1.7) | 14 (19.7) | 3 (2.2) | 13 (16.7) | **<0.001** |
|  |  |  |  |  |  |  |
| **Maternal outcomes** |  |  |  |  |  |  |
| Adverse maternal cardiac outcome | 114 (29.2) | 27 (23.7) | 30 (43.5) | 23 (17.7) | 34 (44.2) | **<0.001** |
| Maternal mortality | 4 (1.0) | 0 (0) | 1 (1.5) | 3 (2.2) | 0 (0) | 0.305 |
| Heart failure | 23 (5.9) | 8 (7.0) | 6 (8.8) | 2 (1.5) | 7 (9.1) | 0.033 |
| Thromboembolic event | 34 (8.6) | 4 (3.4) | 11 (16.2) | 10 (7.5) | 9 (11.7) | 0.018 |
| Hemorrhagic event | 76 (19.5) | 16 (14.0) | 21 (30.9) | 11 (8.5) | 28 (36.4) | **<0.001** |
| Postpartum | 56 (73.7) | 8 (50.0) | 21 (100.0) | 6 (54.5) | 21 (75.0) | **0.001** |
| Endocarditis | 3 (0.8) | 0 (0) | 1 (1.5) | 2 (1.5) | 0 (0) | 0.395 |
| Arrhythmia | 13 (3.4) | 1 (0.9) | 4 (5.9) | 4 (3.1) | 4 (5.2) | 0.176 |
|  |  |  |  |  |  |  |
| **Fetal and neonatal outcomes** |  |  |  |  |  |  |
| Fetal death | 108 (27.1) | 30 (25.4) | 15 (21.7) | 56 (41.8) | 7 (9.1) | **<0.001** |
| Miscarriage <24 weeks | 62 (15.5) | 21 (18.0) | 6 (8.5) | 29 (21.7) | 6 (7.8) | 0.014 |
| Early miscarriage (<14 weeks | 34 (8.5) | 9 (7.7) | 5 (7.1) | 17 (12.7) | 3 (3.9) | 0.144 |
| Late miscarriage (14-23 weeks) | 28 (7.0) | 12 (10.3) | 1 (1.4) | 12 (9.0) | 3 (3.9) | 0.055 |
| Stillbirth (≥24 weeks) | 13 (3.3) | 6 (5.1) | 2 (2.9) | 5 (3.7) | 0 (0) | 0.219 |
| Birth weight, grams, mean ± SD | 2736 ± 544 | 2724 ± 479 | 2656 ± 690 | 2741 ± 459 | 2818 ± 587 | **<0.001** |
| Small for gestational age | 49 (18.4) | 14 (17.1) | 8 (16.3) | 18 (24.3) | 9 (14.5) | 0.493 |
| Congenital heart disease | 5 (1.8) | 1 (1.2) | 1 (2.0) | 1 (1.3) | 2 (3.0) | 0.871 |
| Other congenital disease | 8 (2.9) | 2 (2.4) | 2 (3.9) | 1 (1.3) | 3 (4.6) | 0.645 |
| Neonatal death | 4 (1.4) | 3 (3.4) | 0 (0) | 1 (1.3) | 0 (0) | 0.310 |

Data are presented as n (%) unless otherwise specified, and relate to the number of pregnancies. Percentages are calculated using pairwise deletion. Extent of missing values is reported in Supplementary Table 1.

*Bonferroni: p-values <0.0083 are considered significant.

BMI, body mass index; LMIC, low-or-middle-income country; NYHA, New York Heart Association classification; heart failure, thromboembolic event, hemorrhagic event, endocarditis and arrhythmia); SEF, systemic ejection fraction.

^a^Regime 1: Continue VKA throughout pregnancy

^b^Regime 2: Change to LMWH early in pregnancy

^c^Regime 3: Change to UFH early in pregnancy, switch to VKA in 2^nd^ trimester

^d^Regime 4: Change to LMWH early in pregnancy, switch to VKA in 2^nd^ trimester
